# Electron interactions with the heteronuclear carbonyl precursor H_2_FeRu_3_(CO)_13_ and comparison with HFeCo_3_(CO)_12_: from fundamental gas phase and surface science studies to focused electron beam induced deposition

**DOI:** 10.3762/bjnano.9.53

**Published:** 2018-02-14

**Authors:** Ragesh Kumar T P, Paul Weirich, Lukas Hrachowina, Marc Hanefeld, Ragnar Bjornsson, Helgi Rafn Hrodmarsson, Sven Barth, D Howard Fairbrother, Michael Huth, Oddur Ingólfsson

**Affiliations:** 1 Science Institute and Department of Chemistry, University of Iceland, Reykjavík, Iceland; 2 Physikalisches Institut, Max-von-Laue-Str. 1, Goethe-Universität, 60438 Frankfurt am Main, Germany; 3 Institute of Materials Chemistry, TU Wien, 1060 Vienna, Austria; 4 Department of Chemistry, Johns Hopkins University, Baltimore, Maryland, USA

**Keywords:** dissociative electron attachment, dissociative ionization, electron induced deposition, electron molecule interaction, focused electron beam induced deposition, heteronuclear FEBID precursors, surface science

## Abstract

In the current contribution we present a comprehensive study on the heteronuclear carbonyl complex H_2_FeRu_3_(CO)_13_ covering its low energy electron induced fragmentation in the gas phase through dissociative electron attachment (DEA) and dissociative ionization (DI), its decomposition when adsorbed on a surface under controlled ultrahigh vacuum (UHV) conditions and exposed to irradiation with 500 eV electrons, and its performance in focused electron beam induced deposition (FEBID) at room temperature under HV conditions. The performance of this precursor in FEBID is poor, resulting in maximum metal content of 26 atom % under optimized conditions. Furthermore, the Ru/Fe ratio in the FEBID deposit (≈3.5) is higher than the 3:1 ratio predicted. This is somewhat surprising as in recent FEBID studies on a structurally similar bimetallic precursor, HFeCo_3_(CO)_12_, metal contents of about 80 atom % is achievable on a routine basis and the deposits are found to maintain the initial Co/Fe ratio. Low temperature (≈213 K) surface science studies on thin films of H_2_FeRu_3_(CO)_13_ demonstrate that electron stimulated decomposition leads to significant CO desorption (average of 8–9 CO groups per molecule) to form partially decarbonylated intermediates. However, once formed these intermediates are largely unaffected by either further electron irradiation or annealing to room temperature, with a predicted metal content similar to what is observed in FEBID. Furthermore, gas phase experiments indicate formation of Fe(CO)_4_ from H_2_FeRu_3_(CO)_13_ upon low energy electron interaction. This fragment could desorb at room temperature under high vacuum conditions, which may explain the slight increase in the Ru/Fe ratio of deposits in FEBID. With the combination of gas phase experiments, surface science studies and actual FEBID experiments, we can offer new insights into the low energy electron induced decomposition of this precursor and how this is reflected in the relatively poor performance of H_2_FeRu_3_(CO)_13_ as compared to the structurally similar HFeCo_3_(CO)_12_.

## Introduction

Direct-write technologies using electron beams for nanostructure deposition can surpass the limitations of standard lithography techniques, such as the growth of three-dimensional nanostructures with complex geometries [1–2]. Focused electron beam induced deposition (FEBID) is a powerful technique relying on the decomposition of transiently adsorbed precursors under low vacuum conditions [3]. Different strategies have been used to identify suitable precursors for this process, which relies on electron–molecule interactions rather than the more common thermal fragmentation of precursor species, and various classes of chemical compounds have been considered [4–5] as precursors for FEBID. For instance, metalorganic precursors containing hydrocarbons and chelating ligands can be stable precursors and simple in handling, but these benefits come at the expense of incorporation of large amounts of carbon in the deposits by incomplete decomposition or co-deposition of the liberated ligands. Recent developments demonstrate elegant deposit purification techniques to obtain pure, high quality metals such as Pt and Au by post-growth treatment and in situ injection of water for carbon removal [6–13]. These oxidative processes are suitable for precious metals, while other approaches such as annealing under vacuum [14] and hydrogen atmosphere [15–16] are suitable for metals such as Co. However, alternative precursors for the direct deposition of high-purity compounds are desired especially for non-precious metals and more complex compositions.

In FEBID precursor decomposition is primarily induced by secondary electrons produced as the high-energy primary beam impinges on the substrate's surface [17–18]. These secondary electrons span a wide energy range with significant contribution close to 0 eV, a peak intensity well below 10 eV and a high energy tail extending well above 100 eV (see e.g., [19–21] and references therein). In this energy range fragmentation may be affected by four distinctly different processes, which are active within different energy ranges, and more importantly, lead to distinctly different processes; dissociative electron attachment (DEA), dissociative ionization (DI), and neutral and dipolar dissociation upon electron excitation (ND and DD). An account of the nature of these processes, their energy dependence and the resulting product formation in relation to their role in FEBID is given in a recent review by Thorman et al. [22]. A more general, and detailed account on the nature of these processes can be found in [23–29] and references therein.

Gas phase experiments under controlled single collision conditions, where the incident electron energy may be varied within the relevant range, are ideal to study product formation through the individual processes. Accordingly, such experiments have been used to map the energy dependence of the absolute and relative cross sections for low energy electron induced decomposition of a number of potential and currently used FEBID precursors. These include Co(CO)_3_NO [30–31], Pt(PF_3_)_4_ [32–33], W(CO)_6_ [34], MeCpPtMe_3_ [35], Fe(CO)_5_ [36], and more recently (η^3^-C_3_H_5_)Ru(CO)_3_Br [37–38] and the heteronuclear precursor HFeCo_3_(CO)_12_ [39–40]. However, though such gas phase experiments are well suited to map the extent and energy dependence of the individual processes, their predictive value is limited by the fact that these do not reflect the actual conditions when the precursor molecules are adsorbed on surfaces, as is the case in FEBID. Furthermore, current gas phase experiments rely on the detection of charged fragments, leaving the potentially significant neutral dissociation [22,33,41] upon electron excitation largely unexplored.

The single electron/molecule collision information obtained in the gas phase study may not be sufficient to understand all of the molecular level processes that occur in FEBID, because deposition does not occur through isolated molecules in the gas phase, but on a surface. As a step towards understanding the reactions of adsorbed precursor molecules in FEBID, UHV-surface science studies have been performed, in which nanoscale thin films of precursor molecules adsorbed onto inert substrates were irradiated with 500 eV electrons. Changes in the composition and bonding in the film have been analyzed with X-ray photoelectron spectroscopy (XPS), reflection-absorption IR spectroscopy (RAIRS), and/or high-resolution electron energy loss spectroscopy (HREELS), while mass spectrometry has been used to identify gas phase species generated as a result of electron irradiation. As such the surface science experiments represent an increased level of complexity compared to gas phase experiments, with greater relevance to FEBID. However, such surface studies are conducted in UHV, at low temperatures and under non-steady state conditions and do thus not fully mimic the actual conditions in FEBID.

The surface science approach has nonetheless been proven effective in elucidating electron triggered decomposition of several FEBID precursors including Pt(PF_3_)_4_ [42], W(CO)_6_ [43], MeCpPtMe_3_ [44–45]_,_ Co(CO)_3_NO [46], Fe(CO)_5_ [47] and potential new precursors such as cis-Pt(CO)_2_Cl_2_ [48] and (η^3^-C_3_H_5_)Ru(CO)_3_Br [49]. From these surface science studies, it can be concluded that in general electron induced dissociation of surface adsorbed precursor molecules proceeds in two steps. Electron induced desorption of ligands associated with the precursor occurs to some extent in the first step (e.g., desorption of one of the PF_3_ groups in Pt(PF_3_)_4_ to form a Pt(PF_3_)_3_ surface bound intermediate [42]). In the second step, ligand decomposition typically dominates (e.g., decomposition of the residual PF_3_ ligands in the Pt(PF_3_)_3_ intermediate, the loss of fluorine and the formation of a Pt deposit contaminated by P), although thermal reactions of surface intermediates produced in the initial decomposition step can also be important (e.g., PF_3_ desorption from the Pt(PF_3_)_3_ intermediate if the substrate temperature is sufficiently high [50]).

To date, the most popular precursor class for FEBID is homometallic metal carbonyls of homo- and heteroleptic nature with sufficient vapor pressure. For instance, Fe(CO)_5,_ [51–52] Fe_2_(CO)_9_ [53–54] and Co_2_(CO)_8_ [55] have been shown to yield deposits with high metal content (>60 atom %). In addition, high resolution FEBID of metal nanostructures below 30 nm [56] and successful 3D growth [57] has been demonstrated; however, autocatalytic deposition by spontaneous dissociation on activated surfaces should be avoided for a selective deposition [58–59]. The potential of undesired non-electron induced autocatalytic decomposition illustrates the complexity of the task of identifying precursors yielding high metal content with sufficient stability towards autocatalytic dissociation. Ru_3_(CO)_12_ has been used for FEBID in an earlier report on low temperature substrates [60]; however, the composition of the decomposition product remains unknown and FEBID using a substrate at room temperature could not replicate the earlier results based on chilled substrates [61]. Successful deposition of Ru containing structures has been demonstrated from an organometallic precursor leading to RuC_9_ and required oxygen co-feeding to remove carbon resulting in RuO_2_ [61]. Reports on a heteroleptic Ru carbonyl precursor suggest that the carbonyl ligands can be cleaved more efficiently by low energy electrons than other ligands such as allyl and halides [37,49]. Therefore the investigation of Ru carbonyls as potential FEBID precursors is a promising route. Presently, deposition of heterometallic or composite materials containing more than one metal is usually realized by using multiple injection systems [62–65]. Recently, an alternative strategy based on heterometallic HFeCo_3_(CO)_12_ precursor species has been demonstrated, which allows for direct writing of nanoscale deposits with high resolution, predefined metal ratio and high metal content (>80 atom %) [66]. High purity of deposits and high resolution writing are essential to engineer geometries that are not accessible by crystallization or other template-based approaches. One field of interest in respect to such metallic deposits is the investigation of physical phenomena such as magnetism at the nanoscale. Magnetic nanostructures are fundamental building blocks for applications in data storage and processing as well as the potential successor technologies based on magnonics [67] and spintronics [68] combined with high integration density relying on 3D nanostructure formation. Two- and three-dimensional structures of FEBID-derived magnetic nanostructures have been prepared, [16,53,56–57,69–71] but alternative precursors are desired to predefine different compositions and increase spatial resolution of deposits.

**Figure 1:**
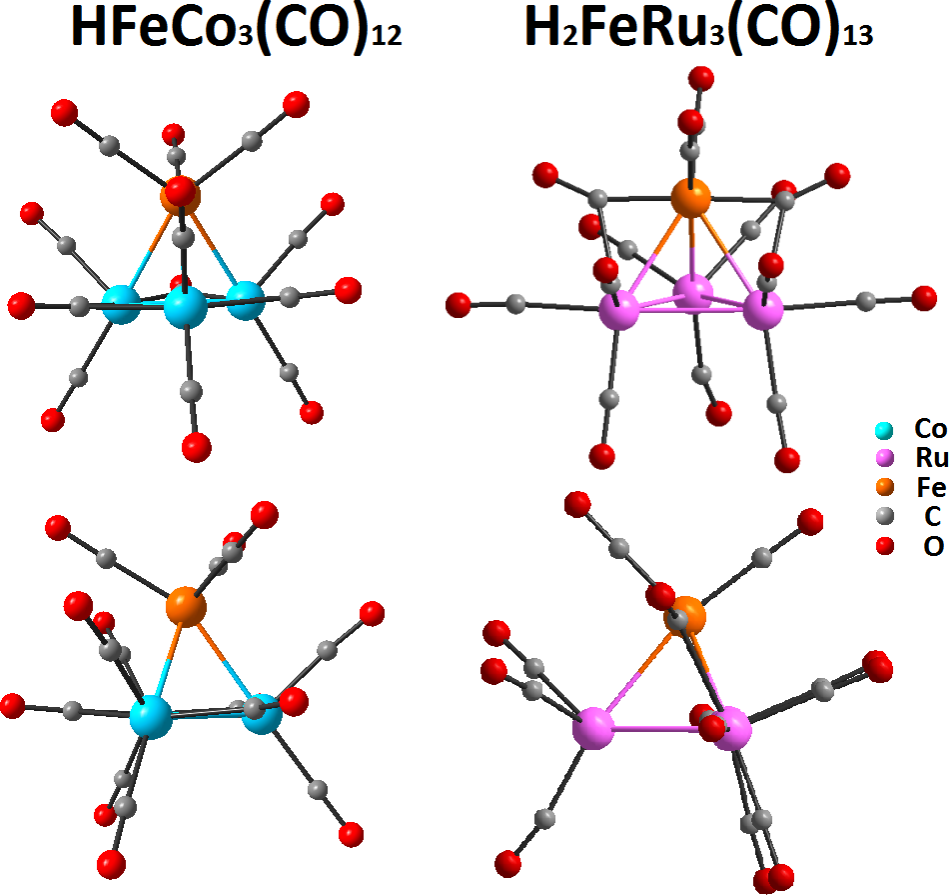
Structural arrangement of HFeCo_3_(CO)_12_ and H_2_FeRu_3_(CO)_13_ illustrating differences in symmetry and ligand bonding. The structures have been drawn using the crystal structure data determined for the two molecules and are shown in two orientations. Hydrogen atoms are omitted because their position cannot be determined by single crystal XRD. Their location is in the center of the Co_3_ basal plane for HFeCo_3_(CO)_12_ [66] and in bridging position between either equivalent Ru atom containing a CO bridge to the Fe apex and the one Ru Atom with exclusively terminal CO ligands in H_2_FeRu_3_(CO)_13_ [72].

The structures of molecular precursor species are required for theoretical treatment and calculation of orbital energies for the electronic ground state (highest occupied molecular orbital; HOMO) and as a base for calculations of the singly occupied molecular orbital (SOMO) energy of the respective anion formed upon electron attachment. Thus, in context to the current discussion, the solid state structures of HFeCo_3_(CO)_12_ and H_2_FeRu_3_(CO)_13_ have been obtained by single crystal X-ray diffraction (Experimental section and as described in literature [66,72]). Figure 1 shows both molecular structures side by side and illustrates the common tetrahedral framework of the metal atoms of these heterometallic clusters.

In H_2_FeRu_3_(CO)_13_ and HFeCo_3_(CO)_12_ each tetrahedron contains one iron atom and three ruthenium or cobalt atoms, respectively. The coordination sphere contains the carbonyl as well as hydride ligands, which results in a highly symmetrical molecule for HFeCo_3_(CO)_12_ and a much less symmetrical arrangement for H_2_FeRu_3_(CO)_13_. For example, there are two non-equivalent Ru positions including one with three terminal CO ligands and two bridging to the two remaining Ru atoms in the plane, while all positions of the Co atoms are equivalent. Moreover, in contrast to exclusively terminal CO ligands on the Fe apex in HFeCo_3_(CO)_12_, the Fe apex in H_2_FeRu_3_(CO)_13_ contains two bridging and two terminal CO ligands. In addition, the bond lengths of the Fe apex to the three remaining metal atoms within the tetrahedron are in the range of 2.538–2.558 Å for HFeCo_3_(CO)_12_ and 2.655–2.705 Å in H_2_FeRu_3_(CO)_13_. One of the Ru–Fe bonds in H_2_FeRu_3_(CO)_13_, which does not contain any bridging CO ligand, is much longer than the other two and therefore it resembles the transition state upon electron capture as described in literature for HFeCo_3_(CO)_12_ [39]. Structural differences will be important for the electron induced decomposition and are discussed vide infra.

In the current contribution, we report on similarities and differences of the heterometallic precursors H_2_FeRu_3_(CO)_13_ and HFeCo_3_(CO)_12_ using several techniques and allowing for comparison between the electron induced decomposition of these compounds in the gas phase, on the surface and during FEBID. The choice of H_2_FeRu_3_(CO)_13_ was motivated by its structural similarities to those of HFeCo_3_(CO)_12_ which, in turn, has proven exceptionally good performance in FEBID of pure, stoichiometric metal alloy structures [66]. Furthermore, the H_2_FeRu_3_(CO)_13_ precursor is the only stable hydridocarbonyl with 1:3 Fe/Ru ratio. Other hydridocarbonyls, such as H_3_FeRu_2_(CO)_13_^−^ are only stable as anions that cannot be converted into neutral molecules. An exception is H_2_Fe_2_Ru_2_(CO)_13_ with an Fe_2_Ru_2_ tetrahedral metal core. However, this compound requires different synthesis conditions and is not expected to be better suited for FEBID. To the best of our knowledge, this is the first extensive report on a heteronuclear precursor providing well-rounded insight into fundamental electron–molecule interactions including electron induced decomposition characteristics in the gas phase and on surfaces as well as its performance in the actual FEBID process. These studies are highly interesting due to the excellent behavior of HFeCo_3_(CO)_12_ in the FEBID process including high metal content, predefined metal ratio and also the high resolution deposition of nanostructures [66]. In contrast, depositions using H_2_FeRu_3_(CO)_13_ have metal content of merely ≈25 atom % and varying metal ratios dependent on process parameters. We relate similarities and specific differences in structure and bonding and compare the fragmentation behavior of both heteronuclear precursors.

## Results and Discussion

### Gas-phase dissociative electron attachment and dissociative ionization of H_2_FeRu_3_(CO)_13_

In the current section we discuss decomposition of the heteronuclear complex H_2_FeRu_3_(CO)_13_ through dissociative electron attachment (DEA) and dissociative ionization and we compare the fragmentation patterns observed to our previous work on HFeCo_3_(CO)_12_. In the energy range from about 0 eV up to about 25 eV DEA to both these potential precursors is characterized by a very rich fragmentation pattern. For HFeCo_3_(CO)_12_ [39–40], 23 distinct, identifiable, negative ion fragments are observed in this energy range, along with the intact molecular anion, and for H_2_FeRu_3_(CO)_13_ 29 fragments are assigned to discrete molecular compositions. Dissociative ionization of these compounds is also extensive with a dominating contribution from sequential CO loss, but also metal–metal bond cleavage and doubly charged cationic fragments are significant in DI of H_2_FeRu_3_(CO)_13_ at 70 eV impact energy.

**Figure 2:**
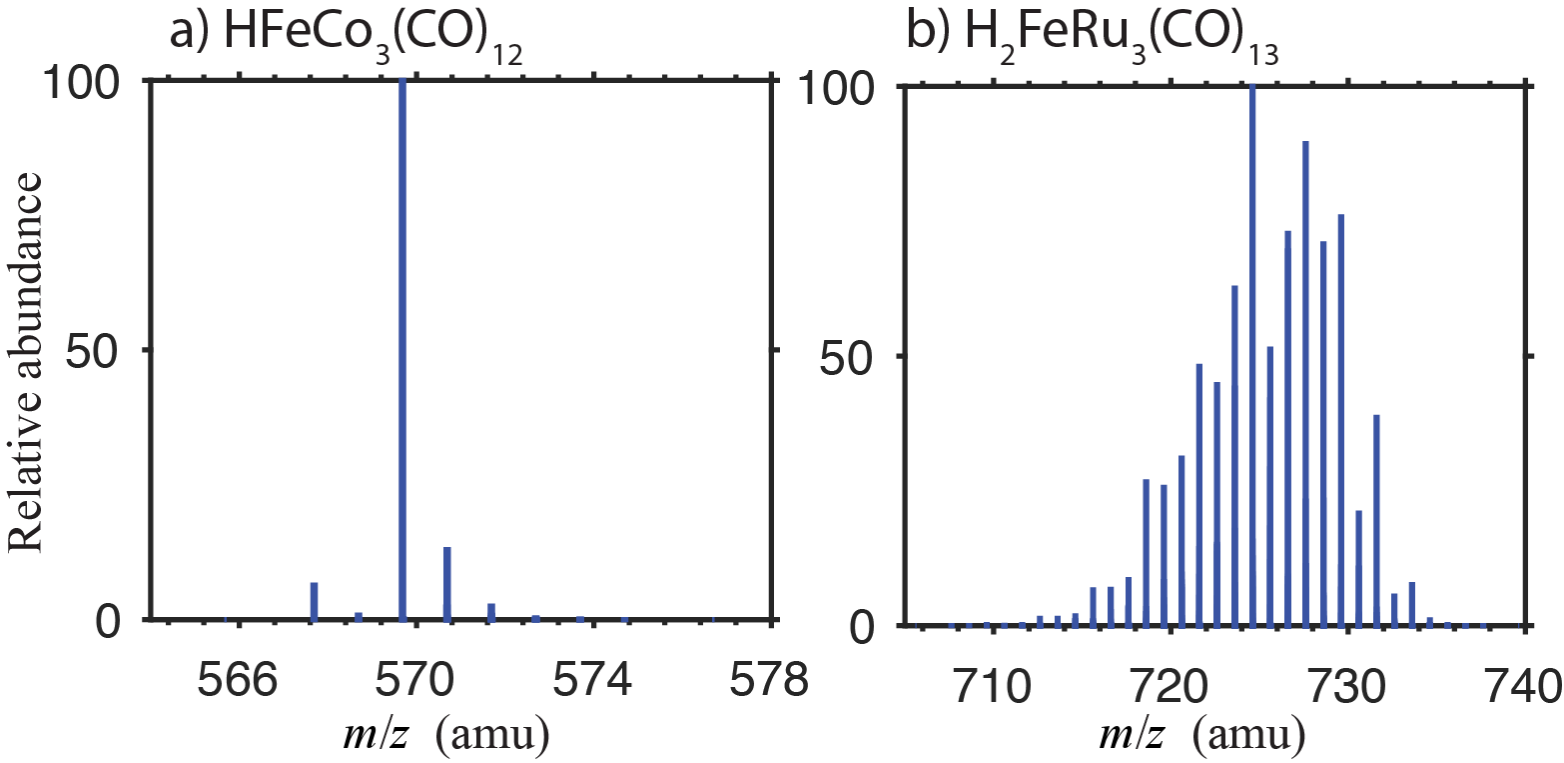
Isotope distribution of a) HFeCo_3_(CO)_12_ and b) H_2_FeRu_3_(CO)_13_. Isotope distribution for both compounds are adapted from [73].

In the current DEA experiments the ion yield curves are recorded by scanning through the relevant electron energy range with the quadrupole mass spectrometer set to only allow transmission of one *m*/*z* ratio. However, to achieve sufficient signal intensity the mass resolution is kept fairly low, practically opening up a transmission window of about 2 mass units. The fragment assignment is fairly straight forward for HFeCo_3_(CO)_12_ where the isotope distribution spans a mass range of 7 amu with one predominant isotope peak. This is to be compared to the mass of CO, i.e., 28 amu, which is the smallest neutral unit lost in the DEA process. For H_2_FeRu_3_(CO)_13_, on the other hand, the isotope distribution spans about 30 mass units with about 10 mass units span of significant peaks. To demonstrate this, Figure 2 compares the isotope distribution for a) HFeCo_3_(CO)_12_ and b) H_2_FeRu_3_(CO)_13_. It is clear from Figure 2 that an unambiguous assignment of contributions to the respective ion yield curves for H_2_FeRu_3_(CO)_13_ from the *m*/*z* ratio alone is often not straightforward. This is further complicated by the fact that the principal mono-isotopic mass of iron is 56 amu, i.e., two times that of CO. Furthermore, DEA cross sections for individual fragments may vary by orders of magnitude and an insignificant *m*/*z* "spill-over" may thus dominate the respective ion yield curves.

To account for this we have calculated the threshold energy for the individual processes at the PBE0 [74–75] /ma-def2 TZVP [76–77] level of theory. We have previously compared the performance of PB86 to that of PBE0 for threshold calculations in DEA to HFeCo_3_(CO)_12_ [39] and found that while PB86 reproduced the structural parameters from the X-ray diffraction (XRD) measurements very well, this functional overestimated the threshold energies significantly, PBE0, on the other hand delivered threshold energies in good agreement with our experimental appearance energies. We thus use the threshold energies calculated at the PBE0 level of theory along with the energy dependence of the fragment formation to assign the contributions in the individual ion yield curves to the respective fragments. Furthermore, to aid the discussion, signal identified as *m*/*z* spill over in the respective ion yield curves are presented in grey to be clearly distinguishable from the principal contributions under discussion. Finally, while we could state with fair confidence where the hydrogen is still attached to negative ion fragments formed from HFeCo_3_(CO)_12_ [39–40] we have no means to verify this for H_2_FeRu_3_(CO)_13_, this also applies to the DI spectra. Generally, we assume that the hydrogens remain attached to the Ru_3_ base plane but in our discussion we do not explicitly account for their whereabouts, except where these are relevant for the calculation of the thresholds for the respective dissociation channels.

**Figure 3:**
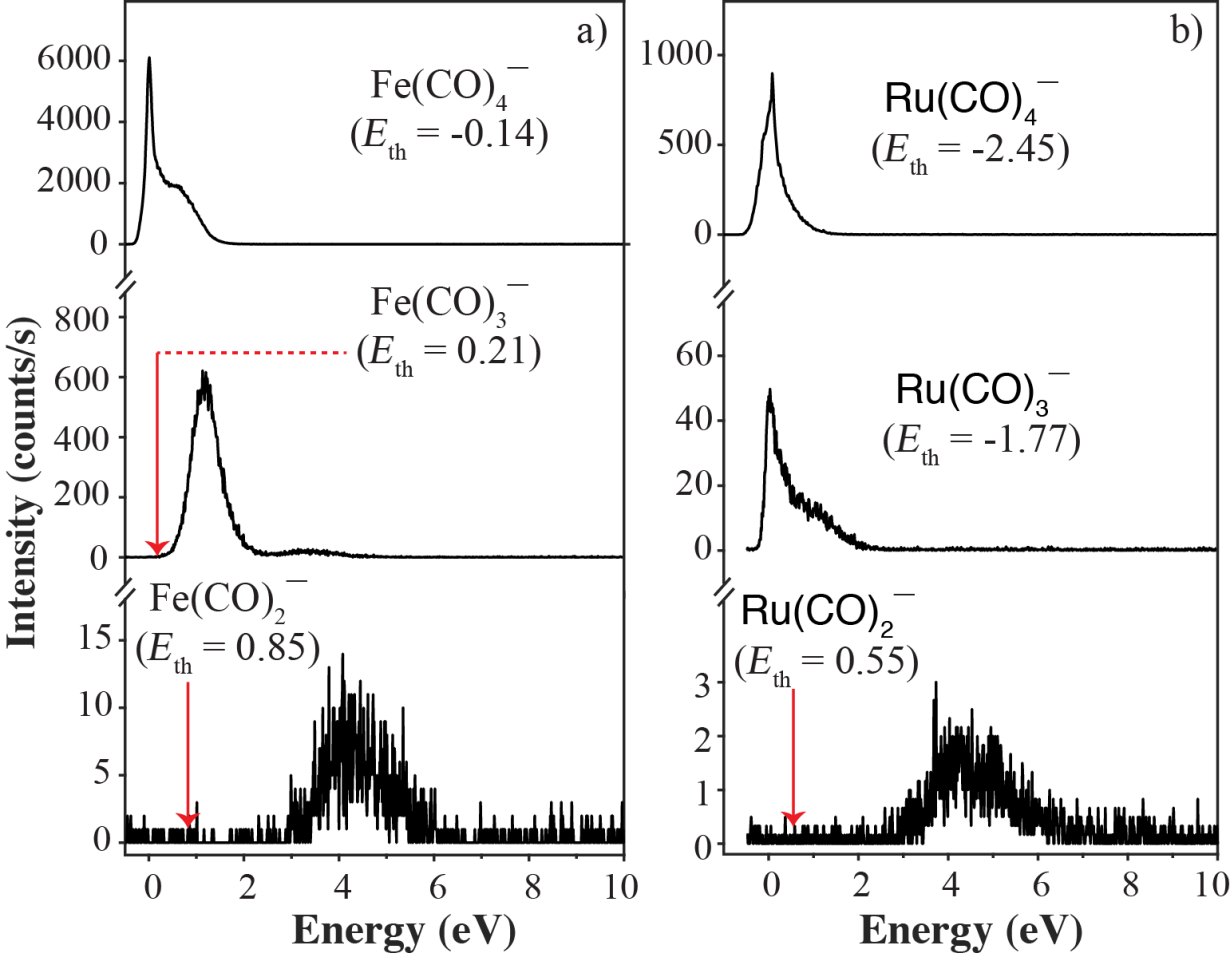
Negative ion yield curves for the formation of a) [Fe(CO)*_n_*]^−^ and b) [Ru(CO)*_n_*]^−^ up on electron attachment in the energy range from 0–10 eV. The thermochemical thresholds for the respective channels calculated at the PBE0/ma-def2 TZVP level of theory are given in parenthesis and indicated by red arrows.

**Figure 4:**
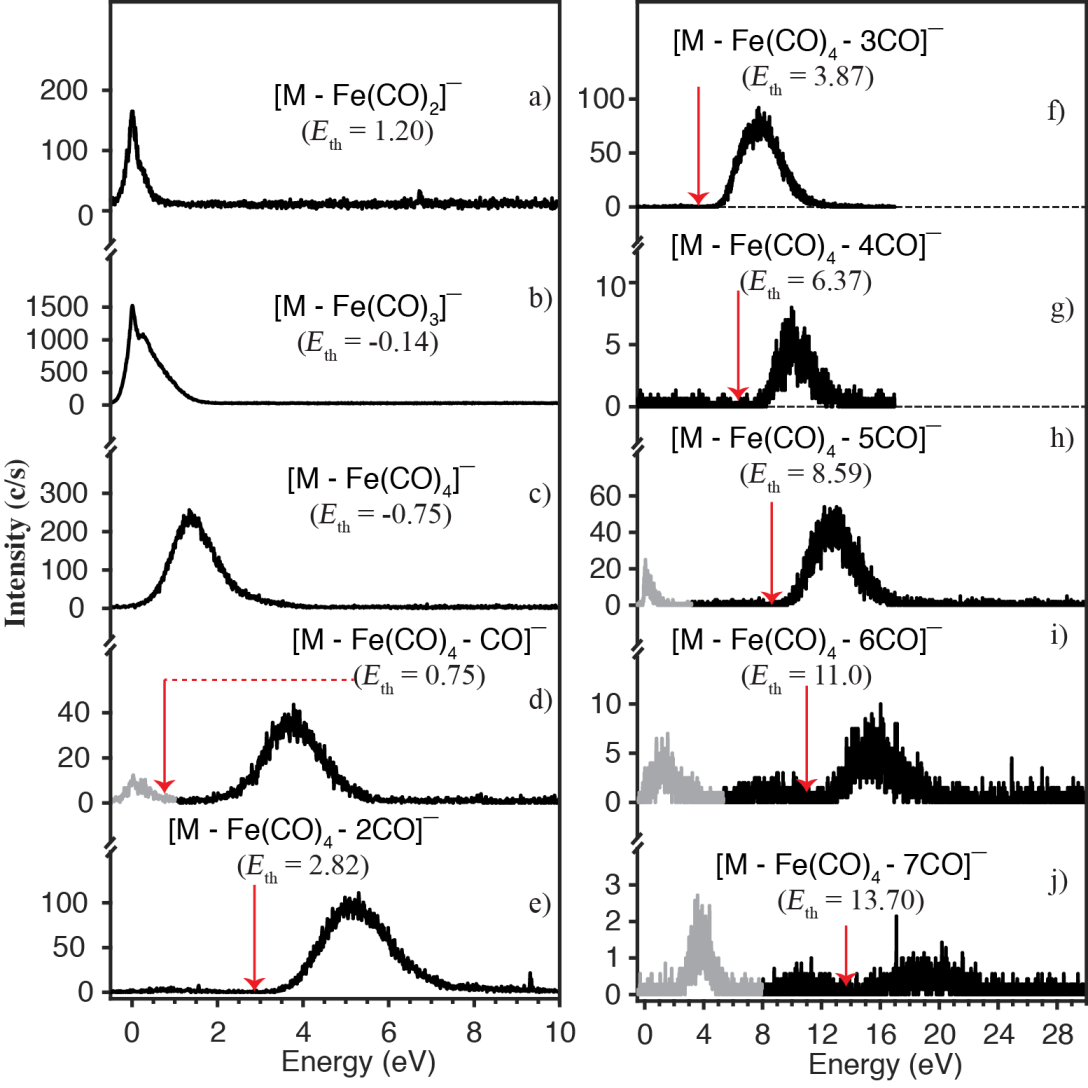
Loss of Fe(CO)_2_ (panel a), Fe(CO)_3_ (panel b), Fe(CO)_4_ (panel c) and additional loss of up to 7 COs (from panel d to panel j) through dissociative electron attachment to H_2_FeRu_3_(CO)_13_. The thermochemical thresholds for the respective channels calculated at the PBE0/ma-def2 TZVP level of theory are given in parenthesis and indicated by red arrows.

**Dissociative electron attachment to H_2_FeRu_3_(CO)_13_:** Dissociative electron attachment to the heteronuclear complexes H_2_FeRu_3_(CO)_13_ is characterized by two primary fragmentation pathways; the apex loss and the loss of a Ru(CO)*_n_*. A further, minor channel leading to the formation of [Ru_2_(CO)*_n_*]^−^ with *n* = 4–7 is also observed. The apex loss appears predominantly with charge retention on the iron containing moiety through the formation of [Fe(CO)_4_]^−^ and to a much lesser extent through the formation of [Fe(CO)_3_]^−^ and [Fe(CO)_2_]^−^, as is shown in Figure 3a. The apex loss also leads to the formation of the complementary fragments [M − Fe(CO)_4_]^−^, [M − Fe(CO)_3_]^−^ and [M − Fe(CO)_2_]^−^ with appreciable intensity on the [M − Fe(CO)_3_]^−^ fragment. Charge retention on the remaining Ru_3_(CO)*_n_* base plane moiety is also observed along with further CO loss, up to 11 CO in total, as shown in Figure 4. Based on our threshold calculations we attribute these channels to the loss of a neutral Fe(CO)_4_ and additional loss of up to 7 CO from the charge retaining moiety.

**Figure 5:**
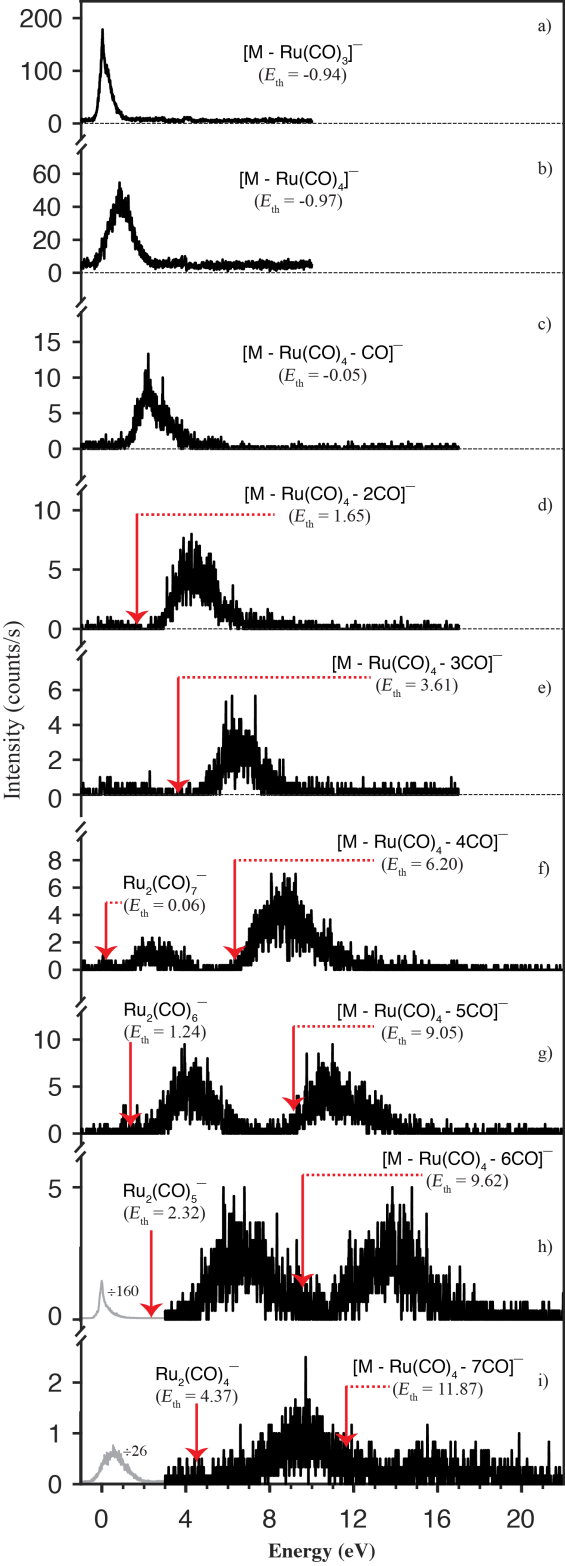
Negative ions formed through loss of Ru(CO)_3_ (panel a), Ru(CO)_4_ (panel b) and further loss of up to 7 COs (panel c to i) through dissociative electron attachment of H_2_FeRu_3_(CO)_13_. Also the ion yields for Ru_2_(CO)_n_ with *n* = 7–4 appear in panels f)–i) respectively, due to the overlap of the isotope distribution of these fragments with that of the respective [M − Ru(CO)_4_ − nCO]^−^ (*n* = 4–7) fragments. The thermochemical thresholds for the respective channels calculated at the PBE0/ma-def2 TZVP level of theory are given in parenthesis and indicated by red arrows.

Similar to the apex loss, we also observe the formation of [Ru(CO)_4_]^−^ with significant intensity and the formation of [Ru(CO)_3_]^−^ and [Ru(CO)_2_]^−^ with considerably less intensity, as shown in Figure 3b. Here we also observe the complementary ions [M − Ru(CO)_4_]^−^ and [M − Ru(CO)_3_]^−^ and further, sequential CO loss from the charge retaining, FeRu_2_ containing moiety up to a total loss of 11 CO units. Similar to the apex loss we attribute these fragments to an initial loss of a neutral Ru(CO)_4_ unit and an additional loss of up to 7 CO units from the charge-retaining moiety. The ion yield curves for these channels are shown in Figure 5.

The respective ion yield curves for the [Fe(CO)*_n_*]^−^ apex loss and the [Ru(CO)*_n_*]^−^ loss from H_2_FeRu_3_(CO)_13_, shown in Figure 3, are almost identical to these observed from HFeCo_3_(CO)_12_ and reported earlier. We have discussed these in detail elsewhere [39]. In brief, based on calculations at the BP86/def2 TZVP level of theory we find the LUMO of HFeCo_3_(CO)_12_ to have a strong Fe–Co antibonding character along the Fe–Co facets and the ground state negative ion formed up on single electron attachment to this molecule relaxes by substantial elongation of two of the three Fe−Co bonds and transformation of one of the terminal Co–COs to a Fe−CO−Co bridging ligand. Furthermore, the relative fraction of the spin density centered on the apical iron in the relaxed ground state [HFeCo_3_(CO)_12_]^−^ anion is markedly larger than that on the cobalt atoms forming the base plane.

**Figure 6:**
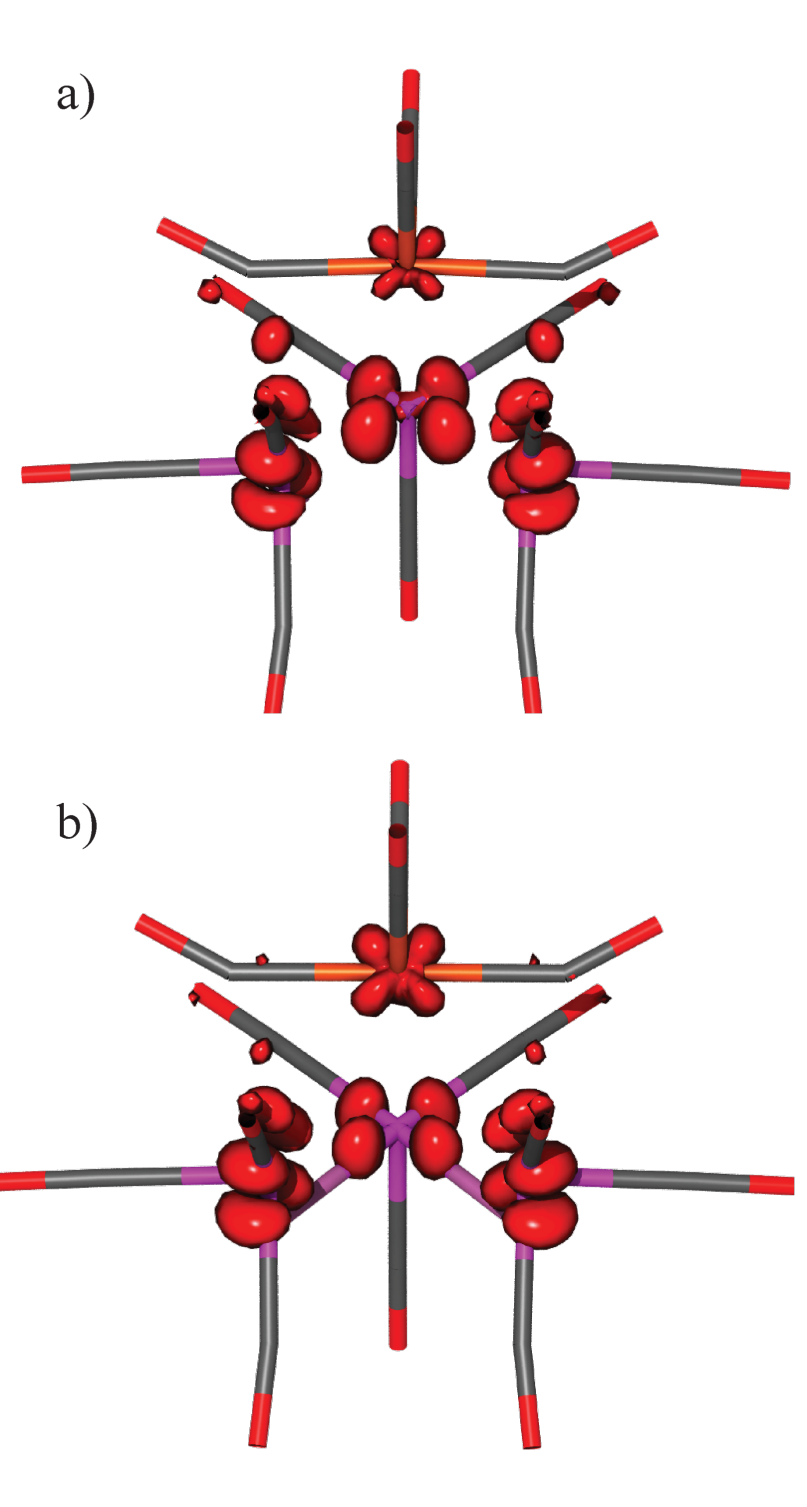
Calculated spin density of the [H_2_FeRu_3_(CO)_13_]^−^ anion; a) in the constrained geometry of neutral H_2_FeRu_3_(CO)_13_ (vertical transition) b) in the relaxed ground state geometry of the anion.

The situation is different for H_2_FeRu_3_(CO)_13_ where the C_3v_ symmetry is broken with a bridging CO between two of the three base plane metal atoms (Ru) and the apex iron. The HOMO of H_2_FeRu_3_(CO)_13_ shows a bonding character along the bridging COs between the base plane and the apex but no significant Ru−Fe bonding contribution is present. Also, the Ru−Fe anti-bonding character of the LUMO is not clear. This is demonstrated in Figure S1, Supporting Information File 1, which shows the isosurfaces for the relevant MOs. Accordingly, single electron occupation of the LUMO of H_2_FeRu_3_(CO)_13_ results in a moderate geometry change as compared to HFeCo_3_(CO)_12_ and the significant weakening of metal–metal bonds from the base plane to the apex observed for HFeCo_3_(CO)_12_ is not observed for H_2_FeRu_3_(CO)_13_. Rather, a moderate metal–metal bond weakening is observed, both within the Ru base plane and between the base plane and the apex, i.e., from 2.934 to 3.037 Å between the hydrogen-bridged rutheniums and from 2.687 to 2.781 Å between the iron and rutheniums, where these are carbonyl bridged. Also a moderate increase in distance between the non-hydrogen bridged rutheniums is observed, i.e., from 2.853 to 2.898 Å, but all further geometry changes are insignificant. (For completeness the geometries of the ground state neutral and anionic H_2_FeRu_3_(CO)_13_, optimized at the BP86/def2-TZVP level of theory, are shown in Figure S4 and the respective Cartesian coordinates and relevant bond lengths and angles are given in Tables S1 and S2, respectively, in Supporting Information File 1). Furthermore, as shown in Figure 6, the spin density calculated for the ground state [H_2_FeRu_3_(CO)_13_]^−^ anion, strained within the neutral geometry, is very similar on all metal atoms. Relaxation to the ground state anionic geometry (Figure 6b), however, leads to a relative increase in the spin density on the Ru base plane atoms as compared to the Fe-apex. It is clear that the comparison of the iso-surfaces for the respective MOs for H_2_FeRu_3_(CO)_13_ and HFeCo_3_(CO)_12_, the spin density of their anions and the geometrical changes between the respective neutral and anionic ground states, does not offer a quantitative explanation of their different behavior with regards to DEA. However, the difference is significant, especially with regards to the relaxation of the metal–metal bonds between the base plane and the apex as well as within the base plane. While the relaxation of the ground state anion of HFeCo_3_(CO)_12_ leads to a spontaneous and significant weakening of the metal bonds from the base plane to the apex, the bond weakening within H_2_FeRu_3_(CO)_13_ is much less significant and is similar within the Ru_3_ base plane and between the base plane and the apex. Furthermore, the relative spin density on the apex iron is much more significant for the anionic ground state of HFeCo_3_(CO)_12_ than for H_2_FeRu_3_(CO)_13_. This is in line with the observation of the apex loss from HFeCo_3_(CO)_12_ being restricted to charge retention on the Fe containing moiety while that from H_2_FeRu_3_(CO)_13_ also leads to a considerable fraction with charge retention on the base plane fragment. Tentatively we offer the explanation that the apex loss from HFeCo_3_(CO)_12_ is a spontaneous process proceeding directly along a repulsive path on the respective potential energy surface of the ground state anion. For H_2_FeRu_3_(CO)_13_, on the other hand, energy dissipation is more effective leading to more apparent competition between the apex loss and base plane fragmentation of the ground state [H_2_FeRu_3_(CO)_13_]^−^ anion.

Furthermore, fragmentation of the base plane is also observed through the formation of [Ru_2_(CO)*_n_*]^−^ with *n* = 4–7, though with comparatively low intensity. The *m*/*z* ratios for the isotope distributions for these fragments overlap considerably with those for [M − Ru(CO)_4_ − *n*CO]^−^ with *n* = 7–4, respectively. These fragments, thus appear in the same ion yield curves displayed in panels (f)–(i) in Figure 5. The assignment of these fragments is based on their calculated thermochemical thresholds, which are displayed in the respective panels. As the [Ru_2_(CO)*_n_*]^−^ *n* = 4–7 thresholds are lower, these could in principle contribute to the corresponding higher energy yields assigned to [M − Ru(CO)_4_ − *n*CO]^−^ with *n* = 7–4, respectively. We do, however, consider this unlikely as the threshold values for the respective fragments correspond very well with the respective onsets. These are distinct progressions of sequential CO loss and as is discussed here below we attribute these to metastable decay. As typical for such processes the onset of a proceeding channel (*n* + 1 CO) coincides with the maximum probability for the preceding one (*n* CO), and correspondingly the onset should coincide with the thermochemical threshold.

Based on our analysis, sequential loss of CO from H_2_FeRu_3_(CO)_13_ with the remaining metal core intact is not observed. This is distinctly different from the fragmentation pattern observed for HFeCo_3_(CO)_12_ through DEA [39–40]. While the apex loss through [Fe(CO)_4_]^−^, [Fe(CO)_3_]^−^ and [Fe(CO)_2_]^−^ formation is also observed from HFeCo_3_(CO)_12_ with similar relative cross sections as for H_2_FeRu_3_(CO)_13_, charge retention on the Co_3_ base plane is not observed in HFeCo_3_(CO)_12_, neither is the formation of [Co(CO)_4_]^−^ or [Co(CO)_3_]^−^. However, similar to H_2_FeRu_3_(CO)_13_ the loss of a single Co and 4–10 CO is observed from HFeCo_3_(CO)_12_, though with very low intensity (see Figure S5, Supporting Information File 1). We attributed this to insignificant neutral Co(CO)_4_ loss associated with further CO loss. Furthermore, sequential CO loss from the intact metal core is the dominant channel in DEA to HFeCo_3_(CO)_12_, while, as stated above, this channel is not observed from H_2_FeRu_3_(CO)_13_.

Figure 4 shows the ion yield curves for the formation of the fragments [M − Fe(CO)_2_]^−^, [M − Fe(CO)_3_]^−^, [M − Fe(CO)_4_]^−^ and [M − Fe(CO)_4_ − *n*CO]^−^ with *n* = 1–7, i.e., the apex loss with charge retention on the Ru_3_ base plane and additional CO loss. The threshold for the corresponding channels are denoted in the respective panels, assuming neutral Fe(CO)*_n_* loss up to *n* = 4 and further sequential CO loss after that. In these calculations the hydrogens are retained on the respective ruthenium base plane fragments. We note in this context, that from the respective *m*/*z* ratios, these fragments could principally also be assigned as [M – (*n* + 2)CO]^−^, however, the threshold for such sequential CO loss from the molecular anion are generally about 3–9 eV above the observed ones. These are listed in comparison with the thresholds for the corresponding [M – Fe(CO)_4_ – *n*CO] fragments in Table S3, Supporting Information File 1.

For H_2_FeRu_3_(CO)_13_ the loss of the neutral Fe(CO)_2_ unit, i.e., the rupture of both Fe–CO bonds to the bridging CO ligands is observed with low intensity through a narrow contribution at around 0 eV (Figure 4a). At the PBE0/ma-def2-TZVP level of theory the threshold for this channel is found to be 1.2 eV, and we thus attribute this low intensity contribution to the high energy tail of the Maxwell–Boltzmann inner energy distribution at the current experimental conditions, *T* = 338 to 343 K. However, we cannot exclude that we have missed the most stable anionic structure in our calculations, despite the consideration of a number of potential structures.

With regards to the charge retention, this is the complementary channel to the formation of [Fe(CO)_2_]^−^ which appearance energy is about 3 eV (Figure 3a) and for which we calculate the threshold to be about 0.85 eV. The next two channels, i.e., the formation of [M − Fe(CO)_3_]^−^ and [M − Fe(CO)_4_]^−^ are found to be exothermic by 0.14 and 0.75 eV, respectively, while the thresholds for the complementary channels leading to the formation of [Fe(CO)_3_]^−^ and [Fe(CO)_4_]^−^ (Figure 3a) are found to be endothermic by 0.21 eV and exothermic by 0.14 eV, respectively. Comparing Figure 3a and Figure 4, it is clear that the ion yield curves for the fragments that are complementary with regards to the charge retention are also complementary with regards to the energy dependence and efficiency of their formation. Hence, while the [M − Fe(CO)_3_]^−^ formation is a dominant channel with a maximum contribution at about 0 eV, the formation of [Fe(CO)_3_]^−^ is observed with moderate intensity and an appearance energy of about 0.5 eV. Conversely, [M − Fe(CO)_4_]^−^ is only observed with moderate intensity and an appearance energy of about 0.5 eV, while the complementary fragment [Fe(CO)_4_]^−^ is the highest intensity fragment observed from this compound, with the main contribution peaking at about 0 eV. In principle all exothermic channels are competing paths at 0 eV incident electron energy, however, the paired energy dependence of the [M − Fe(CO)_3_]^−^ and [Fe(CO)_3_]^−^ fragments as well as that of the [M − Fe(CO)_4_]^−^ and [Fe(CO)_4_]^−^ fragments implies that the rate determining step is strongly coupled to the charge retention. Tentatively we attribute this to two competing initial steps on the respective reaction paths, i.e., the initial rupture of a) a Fe−CO or b) a Ru−CO bond to one of the two Fe−CO−Ru bridging carbonyls. In this picture, the initial rupture of a Fe−CO bond to one of the two Fe−CO−Ru bridging carbonyls leads predominantly to charge retention on the iron containing moiety while a Ru−CO bond rupture leads predominantly to charge retention at the Ru_3_ base plane moiety.

In this context, and to aid the proceeding discussion, we note that the observation window of our experimental setup is about 10 μs, which is the extraction time from the electron–molecule interaction region. Fragments that dissociate further after extraction do not maintain stable trajectories within the quadrupole mass filter and are thus not detected. This is about 50 μs, which is the approximate lifetime required for a fragment to be observed.

We now turn to discuss the [M − Fe(CO)_4_ − *n*CO]^−^ and [M − Ru(CO)_4_ − *n*CO]^−^ fragments from H_2_FeRu_3_(CO)_13_ with *n* = 1–7 (Figure 4d–j and Figure 5c–i), and we compare these with sequential CO loss from HFeCo_3_(CO)_12_ leading to the fragments [M – *n*CO]^−^ with *n* = 3–12. These regressions are remarkable for three different reasons, as is discussed in detail for HFeCo_3_(CO)_12_ elsewhere [40] and we believe that the same considerations hold equally for H_2_FeRu_3_(CO)_13_. In brief, both these molecules show negative ion formation up to above 20 eV incident electron energy, which is more than 10 eV above their respective ionization energy. Furthermore, the lifetime of these ions with regards to autodetachment is long enough to allow for detachment of all CO units from HFeCo_3_(CO)_12_ and Fe(CO)_4_ or Ru(CO)_4_ along with additional loss of up to 7 CO units from H_2_FeRu_3_(CO)_13_. In both cases the formation of individual fragments is confined to a well-defined energy range showing "resonance-like features" in the ion yield curves. The onset of the respective contributions, however, agrees well with their expected thermochemical thresholds and the maxima of [M − *n*CO]^−^ from HFeCo_3_(CO)_12_ and [M – Fe(CO)_4_ – *n*CO]^−^ and [M – Ru(CO)_4_ – *n*CO]^−^ from H_2_FeRu_3_(CO)_13_ coincide with the succeeding [M − (*n* + 1)CO]^−^, [M – Fe(CO)_4_ – (*n* + 1)CO]^−^ and [M – Ru(CO)_4_ – (*n* + 1)CO]^−^ fragments, respectively. This behavior is typical for sequential metastable ligand loss, where [M − *n*CO]^−^ is the precursor of [M − (*n* + 1)CO]^−^ and the extent of the fragmentation is determined by the available excess energy. This, however, would require a quasi-continuous electron attachment over the energy range from around few eV up to above 20 eV for both compounds. For HFeCo_3_(CO)_12_ we postulated [40] that such a continuum is realized through a dense "band*"* of occupied and unoccupied molecular orbitals at the HOMO/LUMO gap of this molecule supporting electron attachment and the formation of long lived, transient negative ions at high energies through multiple electron excitations associated with the attachment process, i.e., the formation of "multi-particle multi-hole resonances". This is enabled through the polynuclear nature of these organometallic compounds providing a dense band of occupied, primarily metal-based orbitals (*d-*orbitals) and the high number and different nature of the carbonyl ligands (bridging and terminal) providing high density of unoccupied ligand CO π* orbitals. Along with the appreciable mixing of these orbitals, this allows for multiple electronic excitations in conjunction with the electron attachment process.

**Figure 7:**
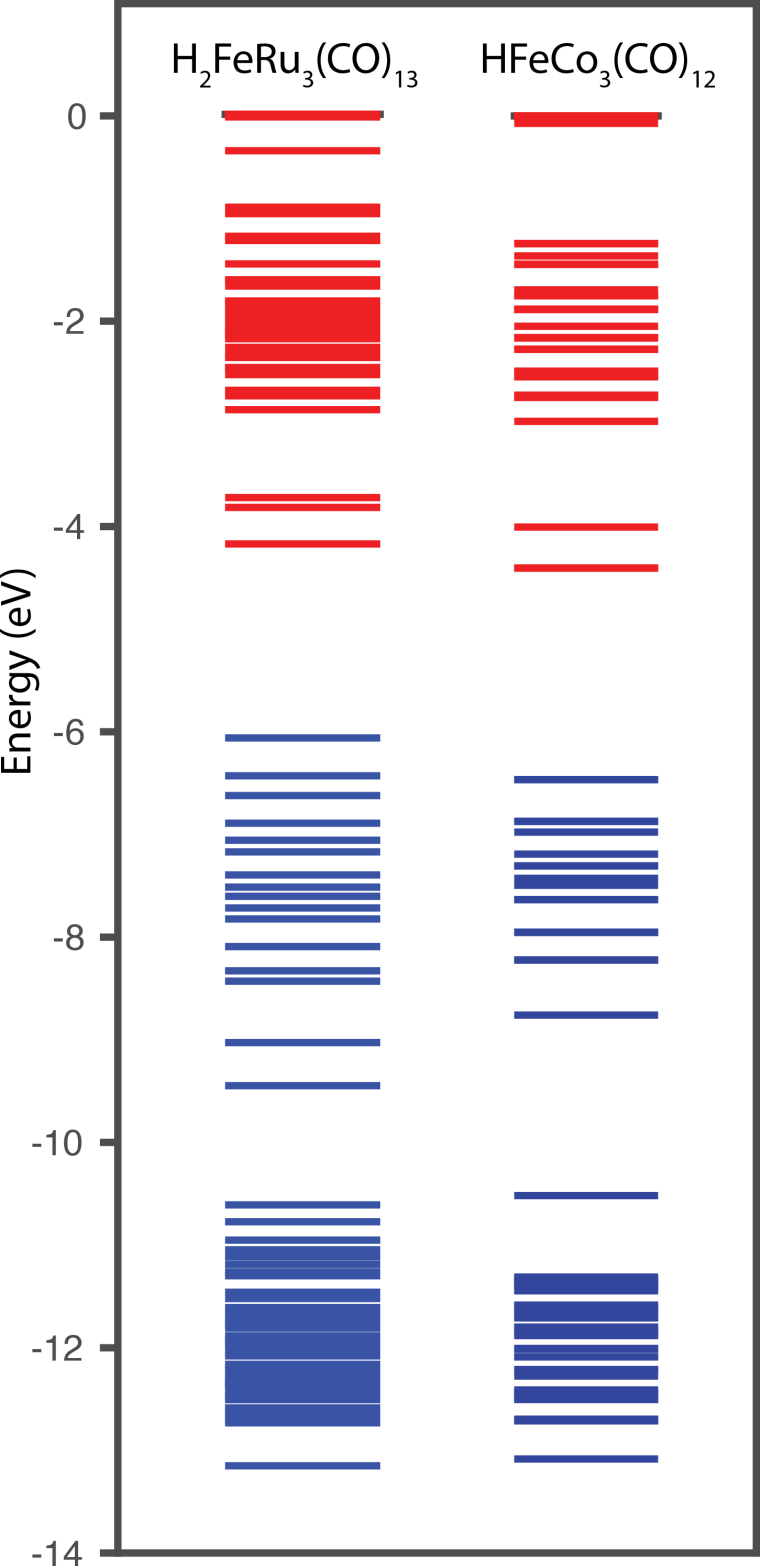
Calculated MO diagrams of H_2_FeRu_3_(CO)_13_ and HFeCo_3_(CO)_12_. Red lines represent the unoccupied molecular orbitals and blue lines the occupied molecular orbitals.

Figure 7 compares the MO diagrams for H_2_FeRu_3_(CO)_13_ and HFeCo_3_(CO)_12_ showing that their MO structure is very similar in this respect, with both compounds possessing a dense band of occupied and unoccupied molecular orbitals at the HOMO/LUMO gap. These are spaced about 3 eV apart allowing for more than 6 electronic transitions at about 20 eV incident electron energy.

In an intermediate extraction, we can conclude that the compounds H_2_FeRu_3_(CO)_13_ and HFeCo_3_(CO)_12_ show a very similar electron attachment profile with a series of two to three low energy single particle resonances supporting negative ion formation in the energy range from 0 to about 2–3 eV. At intermediate energies the MO-structure of these compounds allows for negative ion formation supported through concomitant electronic excitation, i.e., one-hole two-particle resonances. At high energies up to about 20 eV, we anticipate that long lived negative ion formation is supported by multiple electron excitations, i.e., through "multi-particle multi-hole resonances" [40]. Together these resonances provide a quasi-continuous attachment profile from about 0 eV up to above 20 eV.

The main difference between these compounds, with regards to DEA, lies in the fragmentation process of the molecular anions formed, rather than the initial electron attachment process. For HFeCo_3_(CO)_12_ the two main channels are (i) the apex loss leading mainly to the formation [Fe(CO)_4_]^−^ but also [Fe(CO)_3_]^−^ and (ii) sequential CO loss from the molecular anion leading to the fragments [M – *n*CO]^−^ with *n* = 1–12. Hence, there are two parallel paths where the initial CO loss competes with the apex loss in the low energy range:

[1]
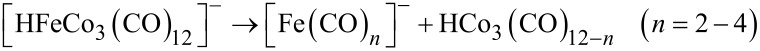


and

[2]
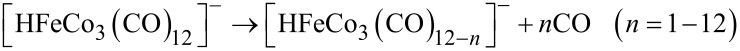


For H_2_FeRu_3_(CO)_13_, on the other hand, the apex loss (mainly as Fe(CO)_4_) or the loss of a single ruthenium from the base plane (mainly as Ru(CO)_4_) precedes all further fragmentation. Where the charge retention is on the metal tetracarbonyl (M(CO)_4_) fragment, further fragmentation of the neutral fragment is not expected, as this channel proceeds predominantly at or close to 0 eV. However, when the charge retention is on the respective Ru_3_ or FeRu_2_ containing fragments further loss of up to 7 CO units is observed. This situation is shown in Equation 3 and Equation 4 for the apex loss as Fe(CO)_4_ and further CO loss from the Ru_3_ base plane fragment:

[3]
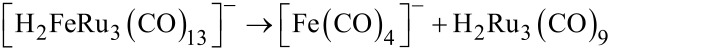


and

[4]
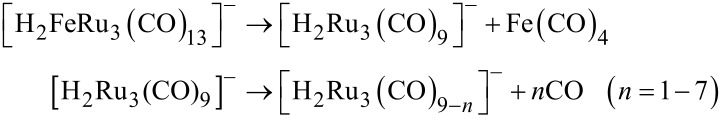


Furthermore, while insignificant base plane fragmentation is observed for HFeCo_3_(CO)_12_, base plain fragmentation of H_2_FeRu_3_(CO)_13_ is observed through [Ru(CO)*_n_*]^−^ and [M – Ru(CO)*_n_*]^−^ formation with *n* = 2–4, [M – Ru(CO)_4_ – *n*CO]^−^ with *n* = 1–7 and [Ru_2_(CO)*_n_*]^−^ with *n* = 4–7.

**Dissociative ionization**, different from DEA, is a non-resonant process with an onset at or slightly above the ionization limit of the respective compounds. At threshold, DI is generally characterized by single bond ruptures, i.e., the lowest energy channels. With increasing electron impact energy further channels open up and the DI cross sections for individual channels increases until the total cross section reaches a maximum in the range between 70 and 100 eV. At higher electron impact energies the energy transfer efficiency diminishes, reflected in a gradual decrease in the total cross section as the impact energy increases further. At about 70 eV all DI channels are generally close to their maxima and DI spectra at this energy normally give a good picture of the integral efficiency of the individual channels, though they do not accurately reflect the onset region where different channels are opening up and the branching ratios are markedly different.

**Figure 8:**
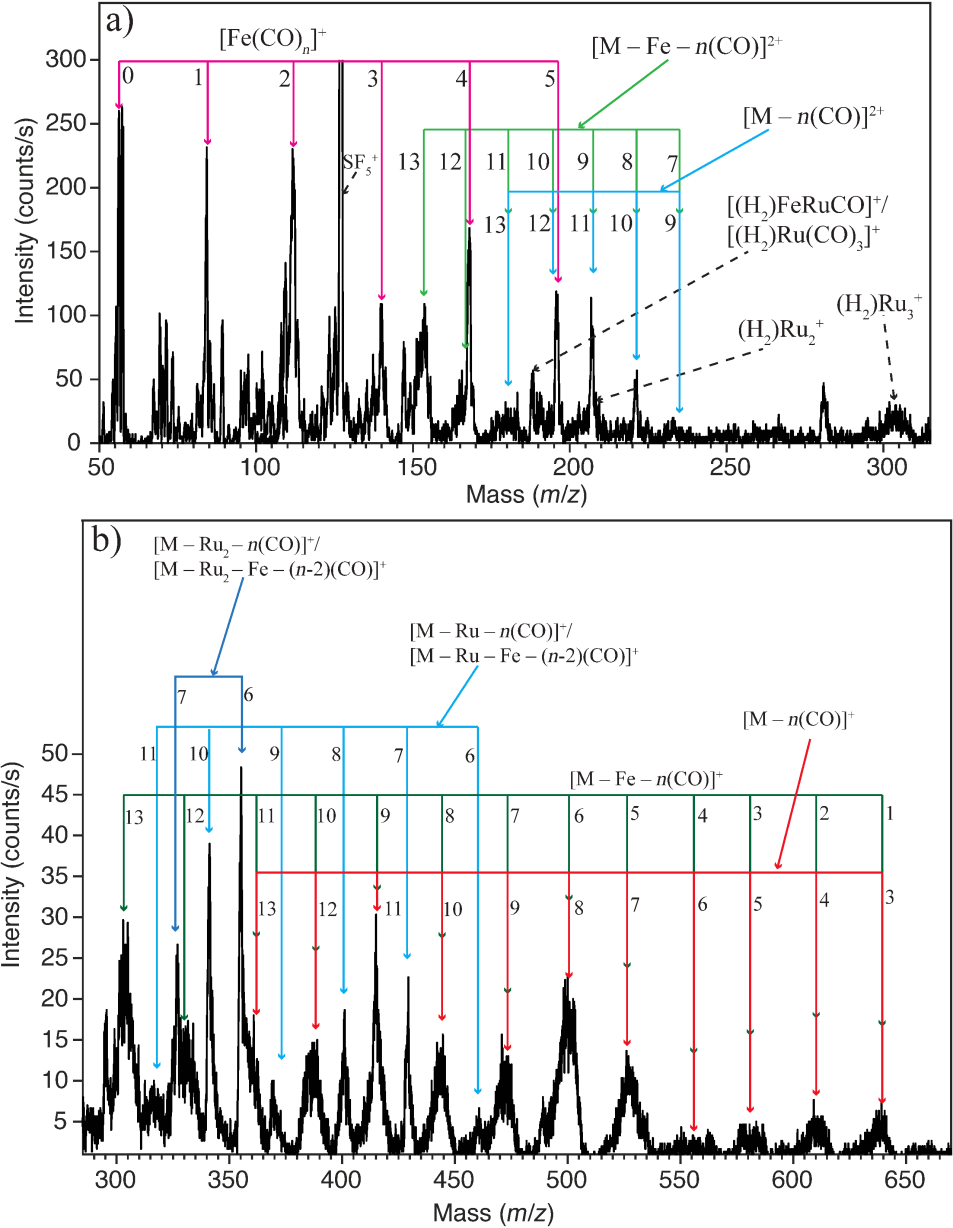
Electron impact ionization spectra of H_2_FeRu_3_(CO)_13_ recorded at electron energy of 70 eV, upper panel shows the positive ion fragments formed in the mass range 50 to 315 amu and the lower panel shows positive ion fragments in the mass range 280 to 670 amu. The label M in the figure is used for H_2_FeRu_3_(CO)_13_.

Figure 8 shows DI spectra of H_2_FeRu_3_(CO)_13_ recorded at an impact energy of 70 eV. Panel (a) shows the *m*/*z* range from about 50 to 315 while panel (b) shows the *m*/*z* range from about 280 to 670. The fragmentation of H_2_FeRu_3_(CO)_13_ at 70 eV impact energy is very rich and characterized by broad contributions and significant overlap resulting from the wide isotope distribution of ruthenium. The accurate interpretation of the spectra is further complicated due to the fact that the mass of the principal iron isotope (56 amu) is two times that of CO, not allowing for differentiation between Fe loss and the loss of two CO from the *m*/*z* ratios alone. Furthermore, in the lower *m*/*z* range we observe contributions from doubly charged fragment ions, though with comparably low intensity. For the low *m*/*z* range up to about 300, the dominating regression can be unambiguously assigned to [Fe(CO)*_n_*]^+^ with *n* = 0–5. The higher *m*/*z* range, on the other hand, is characterized by two main regressions which cannot be unambiguously assigned to defined molecular composition from the *m*/*z* ratios alone. The first regression may be assigned as [M – *n*CO]^+^ with *n* = 3–13, but may also be attributed to [M – Fe – (*n* − 2)CO]^+^. The second regression is [M – Ru – *n*CO]^+^ with *n* = 6–11 which similarly may also be attributed to [M – Ru – Fe – (*n* − 2)CO]^+^. Further significant contributions are observed from [M – 2Ru – 6CO]^+^ and [M – 2Ru – 7CO]^+^ in this *m*/*z* range. Again, these *m*/*z* fragments may also be assigned to the respective [M – 2Ru – Fe – 4CO]^+^ and [M – 2Ru – Fe – 5CO]^+^ ions.

To enable better comparison with the surface experiments discussed in the next section and specifically to try to identify whether DEA or DI is likely to play the dominating role in the decomposition of H_2_FeRu_3_(CO)_13_ physisorbed on a substrates surface, we have estimated the average CO loss per incident electron for both the DI and DEA process. For DEA this is estimated by multiplying the integrated intensity of the individual channels with the number of CO lost in the process, summing this up for all channels and dividing the derived total CO loss with the total DEA intensity. For DI the same procedure is used, however, the respective integral intensities are estimated from the peak intensities at the *m*/*z* ratios for the respective principal isotopes. The measured intensities are then divided by the fractional contribution of the principal isotope to the total isotope distribution. For simplification only the isotope distribution of the metal content of the respective fragments is considered.

The main problem with these estimations is that we do not have any information on the fragmentation of the neutral counterparts; this is especially true for DI where we have no information on the available excess energy.

To account for this, we have calculated a lower limit and a higher limit for the CO loss from H_2_FeRu_3_(CO)_13_ per incident, both for the DI and the DEA process. For the lower limit in DI we presume the high *m*/*z* regressions to be associated with neutral iron loss as neutral Fe(CO)_4_, i.e., loss of the apex iron with both the bridging carbonyls and both terminal carbonyls. For the [Fe(CO)*_n_*]^+^ regression we presume that the neutral counterpart stays intact. For the higher limit we presume that the high *m*/*z* ratios are not associated with iron loss and that the neutral counterparts to the [Fe(CO)*_n_*]^+^ regression fragment through complete CO loss. Similarly, for DEA we estimate the upper limit by assuming additional CO loss from the neutral counterparts formed in the individual processes. However, here we have a fair estimation of the excess energy available as we have calculated the thermochemical thresholds for the individual processes. For the neutral Fe(CO)_4_ and Ru(CO)_4_ loss and additional CO loss the onset of the individual contributions is mostly close to the calculated thermochemical threshold of the individual processes, but the respective contributions generally stretch over a range of about 4–5 eV. On the high energy side of the respective contribution, further loss of 2–3 CO from the respective metal neutral tetracarbonyl is thus in principle possible. Accordingly, we calculate the lower limit for CO loss through DEA by presuming the intact neutral Fe and Ru carbonyls (mainly tetracarbonyls). For the higher limit we simply presume additional loss of two CO from these.

From these estimations we derive the bracketing numbers 0.5–3 for CO loss from H_2_FeRu_3_(CO)_13_ per incident through DEA and 3–9 for DI. In this context we note that all DEA channels are associated with metal–metal bond ruptures, while in DI this number is somewhere between 50–100% depending on how large a fraction of the *m*/*z* ratios matching the [M – *n*CO]^+^ regression are actually due to the formation of [M – Fe – (*n* − 2)CO]^+^. For HFeCo_3_(CO)_12_ the same estimations give the bracketing numbers 4–9 for CO loss per incident through DI and 2–3 for DEA, while metal–metal bond rupture constitutes 50% of the DI intensity and about 30% of the DEA intensity.

Finally, we emphasize that we are only able to account for DEA and DI in the current experiments and we are blind to all fragmentation caused by neutral dissociation upon electron excitation. For Pt(PF_3_)_4_, it has been shown that the cross sections for electronic excitations are very significant [33] and it is reasonable to assume that the cross sections for such fragmentation is comparable to the fragmentation observed in DEA. This assumption is derived from the notation that the underlying electronic excitations correspond to the respective resonances observed in DEA, i.e., a single particle resonance in DEA has a corresponding one-hole one-particle resonance in electronic excitation and the same is true for core excited one-hole two particle DEA resonances as well as the postulated multi-particle resonances recently discussed in conjunction with CO loss from HFeCo_3_(CO)_12_ through DEA [40]. In fact, in a theoretical study of the excited states observed in the electron energy loss study on Pt(PF_3_)_4_ [33], many of these states have been shown to be dissociative, indicating a high ND efficiency for this molecule [41].

### Electron induced surface reactions of H_2_FeRu_3_(CO)_13_

The surface reactions of adsorbed H_2_FeRu_3_(CO)_13_ molecules were studied under UHV conditions (*P*_base_ < 4 × 10^−9^ mbar). Ultra-thin (<2–3 nm) H_2_FeRu_3_(CO)_13_ films were deposited onto a cooled, sputter-cleaned Au substrate before being exposed to 500 eV incident electrons generated by a commercial flood gun. The effect of electron irradiation on the adsorbed H_2_FeRu_3_(CO)_13_ molecules as monitored in situ by X-ray photoelectron spectroscopy (XPS) and mass spectrometry.

**Figure 9:**
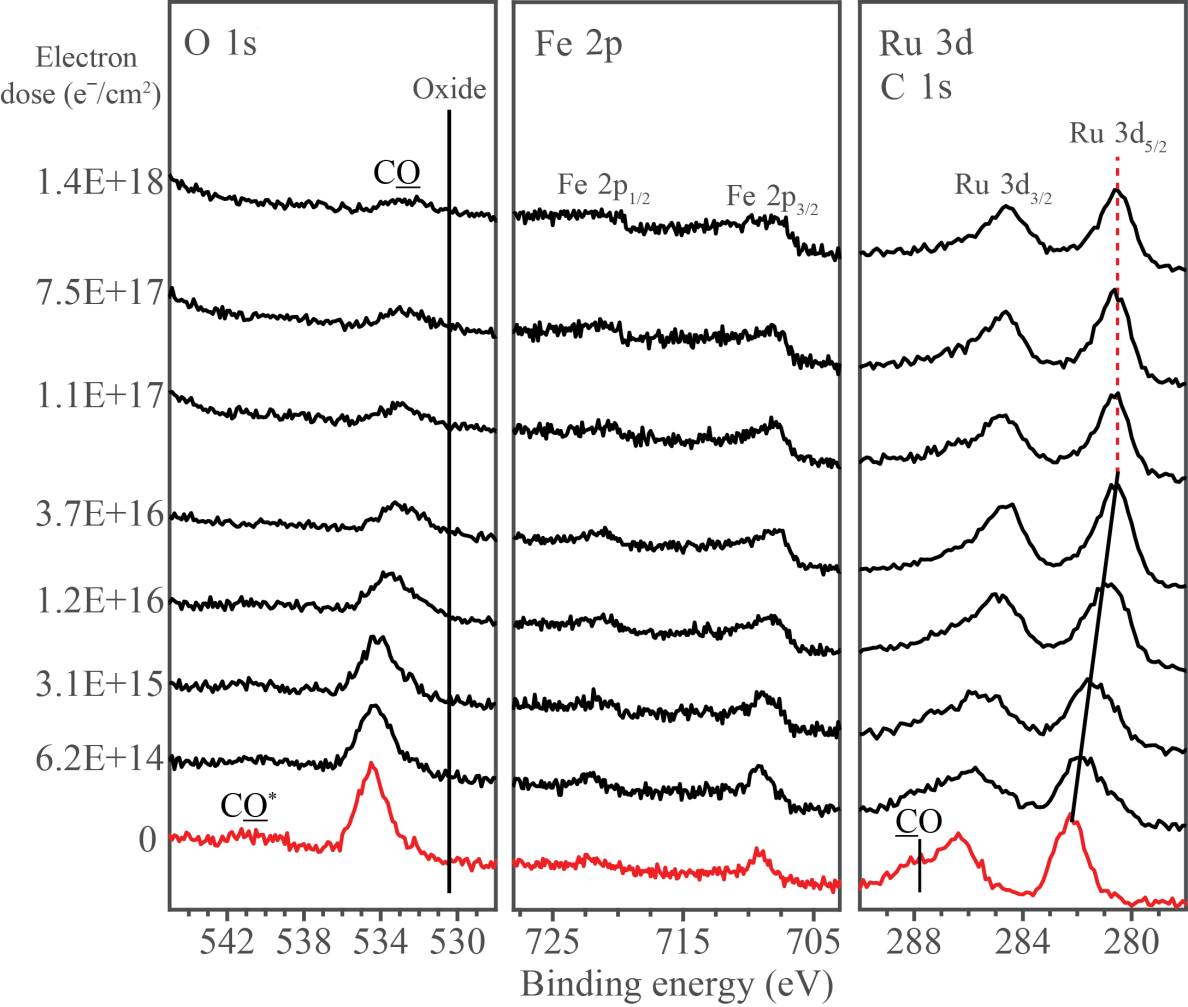
Evolution of O 1s, Fe 2p and Ru 3d/C 1s XPS regions of a H_2_FeRu_3_(CO)_13_ film exposed to electron doses up to 1.4 × 10^18^ e^−^/cm^2^.

Figure 9 shows the evolution of the O 1s, Fe 2p and Ru 3d/C 1s XPS regions of a nanometer-thick film of H_2_FeRu_3_(CO)_13_ adsorbed onto a gold substrate at 213 K, plotted as a function of increasing electron dose. Area analysis reveals that prior to electron irradiation, the O/Ru ratio is ≈4.8 and the O/Fe ratio is ≈14.5. These measured O/Ru and O/Fe ratios, in addition to the absence of any peaks in the O 1s or C 1s regions that would be indicative of CO decomposition, support the idea that upon deposition at 213 K the precursor is molecularly intact prior to electron irradiation.

Prior to electron irradiation, the O 1s region consists of two peaks centered at 534.5 and 540.6 eV, which can be ascribed to the O 1s peak and the higher binding energy/lower intensity π–π* shake up features associated with CO ligands, respectively [78–79]. Electron irradiation produces a significant decrease in intensity within the O 1s XPS region for electron doses <≈5 × 10^16^ e^−^/cm^2^, although thereafter it remains relatively constant in peak position and intensity. During the course of irradiation there is no evidence of oxide formation, which would be expected to produce an asymmetric profile with a peak position at ≈530.5 eV [80].

In the Fe 2p region two peaks are observed prior to electron irradiation, which correspond to the Fe 2p_3/2_ and Fe 2p_1/2_ transitions of H_2_FeRu_3_(CO)_13_, centered at 709.1 eV and 722.3 eV respectively. Upon electron irradiation, there is a decrease in the Fe 2p peak energy (≈0.6 eV) accompanied by a change in the spectral profile from a fairly symmetric peak, indicative of iron atoms in a molecular entity such as H_2_FeRu_3_(CO)_13_, to a peak shape more indicative of metallic iron, likely caused by an increase in the degree of metal-metal bonding between fragments formed by the dissociation of the precursor and/or between fragments and the substrate. Careful analysis of the Fe 2p region reveals that although there is a change in the peak shape there is no statistically significant change in the integrated area of the Fe 2p peaks.

In the Ru 3d/C 1s region, the spectral envelope can be fit with three peaks prior to electron irradiation (fitting can be seen in Figure S2, Supporting Information File 1). Peaks at 286.2 eV and 282.2 eV correspond to the Ru 3d_3/2_ and Ru 3d_5/2_ transitions respectively, while another peak centered at 287.7 eV which appears as a shoulder to the higher binding energy side of the Ru 3d_5/2_ peak can be ascribed to the C 1s peak of the CO ligands [81]. It should be noted that the π–π* shake up feature in the C 1s peak for CO ligands expected at ≈293.2 eV was not observed due to its low intensity. Upon electron irradiation, the Ru 3d_3/2_ and Ru 3d_5/2_ peak positions shift measurably towards a lower binding energy, and the CO peak decreases in intensity. Spectral fitting of the Ru 3d/C 1s XPS region measured after an electron irradiation of a dose of 1.4 × 10^18^ e^−^/cm^2^ reveals an absence of any graphitic carbon (peak position 284.5 eV). As a consequence of electron irradiation the Ru 3d_5/2_ peak shape changes in a fashion analogous to that observed for the Fe 2p peaks without any change in the integrated area of the Ru peaks. Thus, electron irradiation does not cause any desorption of metal from the adsorbed H_2_FeRu_3_(CO)_13_ film at these low temperatures (≈213 K).

During XPS analysis, secondary electrons will be generated and can also cause changes to the adsorbate molecules. As a result, separate control studies were conducted to determine the effect of X-ray irradiation alone on the adsorbate layer. Results from these experiments are shown in Figure S3, Supporting Information File 1, where a H_2_FeRu_3_(CO)_13_ film was continuously exposed to X-rays and simultaneously analyzed using XPS. Comparison with electron irradiated films reveal that X-ray irradiation produces the same changes as electron irradiation, but at a much slower rate. Based on the variation in the O 1s area we estimate that the time taken to acquire one XPS scan of the O 1s, Fe 2p and C 1s/Ru 3d regions corresponds to an electron dose of ≈6.3 × 10^14^ e^−^/cm^2^. Consequently, the effect of X-ray irradiation during the experiments described in Figure 9 is minimal except for the lowest electron doses, where the measured dose based on electron irradiation alone is somewhat underrepresented.

**Figure 10:**
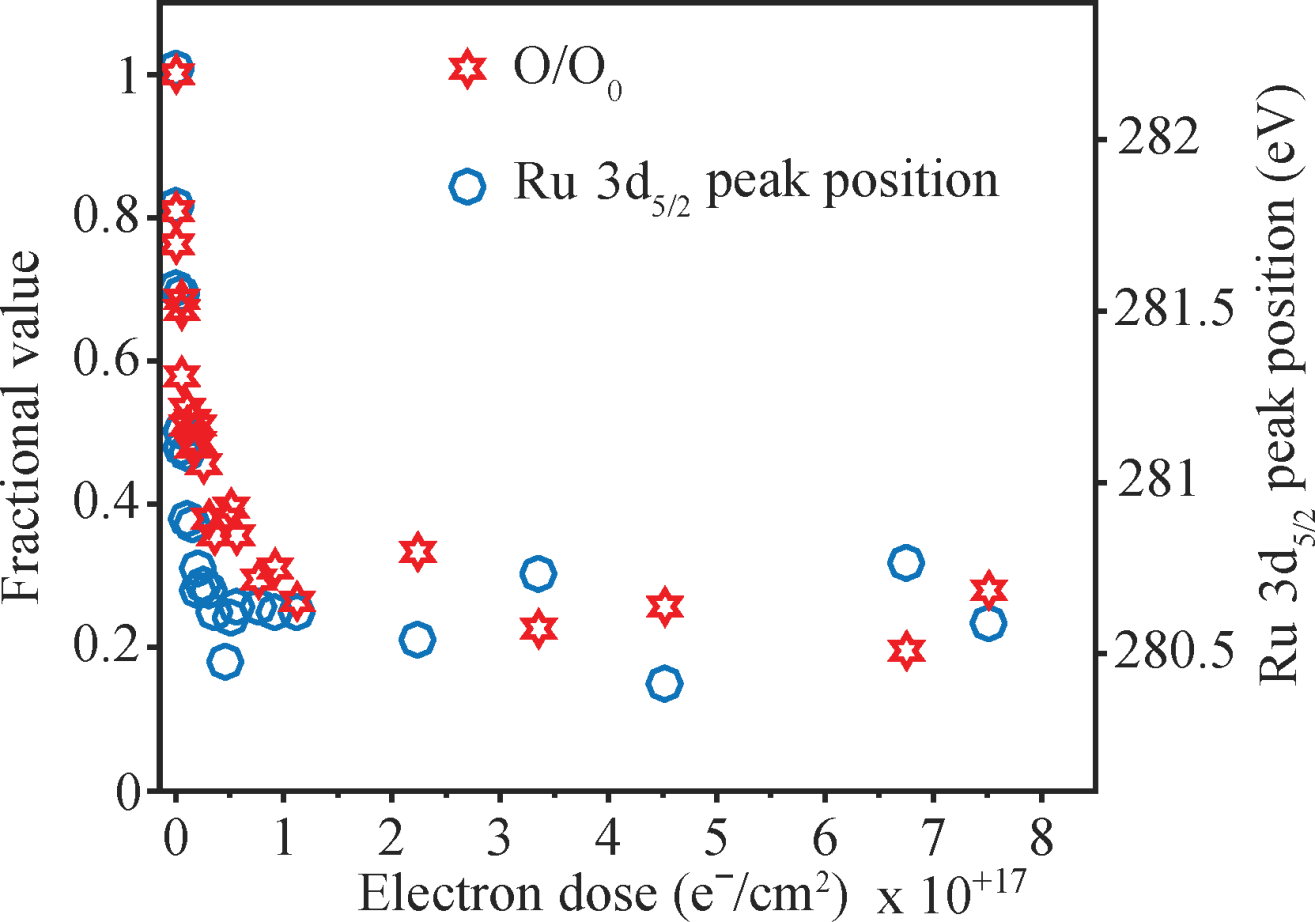
Change in fractional coverage of oxygen atoms (red stars) and, Ru 3d_5/2_ peak position (blue open circle) for H_2_FeRu_3_(CO)_13_ films. Both are plotted as a function of electron dose.

Figure 10 shows that the fractional decrease in the coverage of oxygen atoms and the change in Ru 3d_5/2_ binding energies both follow a similar dependence on the electron dose; specifically, they both decrease significantly for comparatively low electron doses (<≈6 × 10^16^ e^−^/cm^2^), but remain constant thereafter. In the case of the Ru 3d_5/2_ peak the binding energy decreases systematically from 282.2 eV initially to 280.6 eV (close to the Ru metal binding energy of 280.1 eV) after an electron dose of ≈6 × 10^16^ e^−^/cm^2^, implying a partial reduction of Ru in the decomposition process. Over the course of the same electron dose the fractional coverage of oxygen atoms is reduced by ≈70% of its initial value.

**Figure 11:**
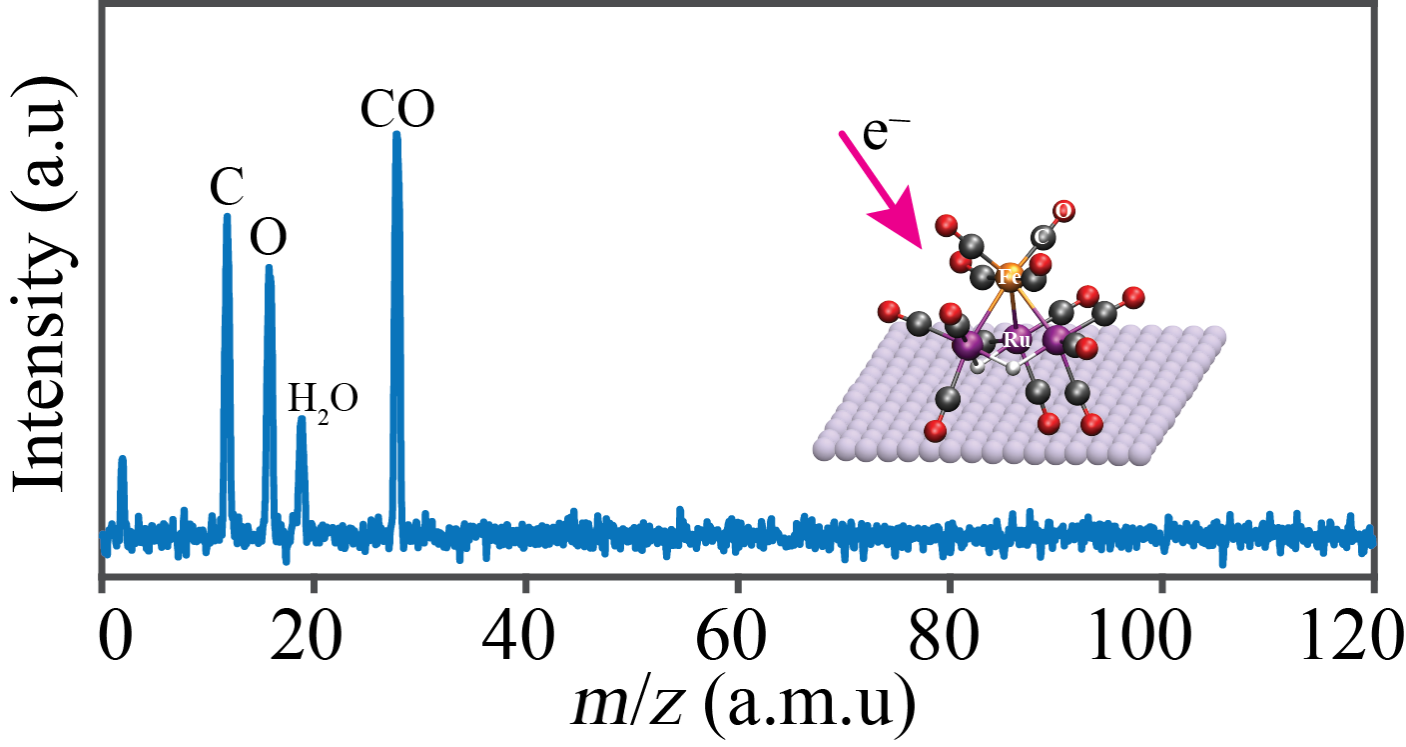
Mass spectrum of neutral gas phase species desorbed from an H_2_FeRu_3_(CO)_13_ film during the course of an electron dose ≈1.2 × 10^17^ e^−^/cm^2^.

Figure 11 shows the mass spectrum recorded during the electron irradiation (dose ≈1.2 × 10^17^ e^−^/cm^2^) of a H_2_FeRu_3_(CO)_13_ film. The only signals observed were at *m*/*z* 12 (C), 16 (O) and 28 (CO), along with some hydrogen and residual water vapor in the UHV chamber.

**Figure 12:**
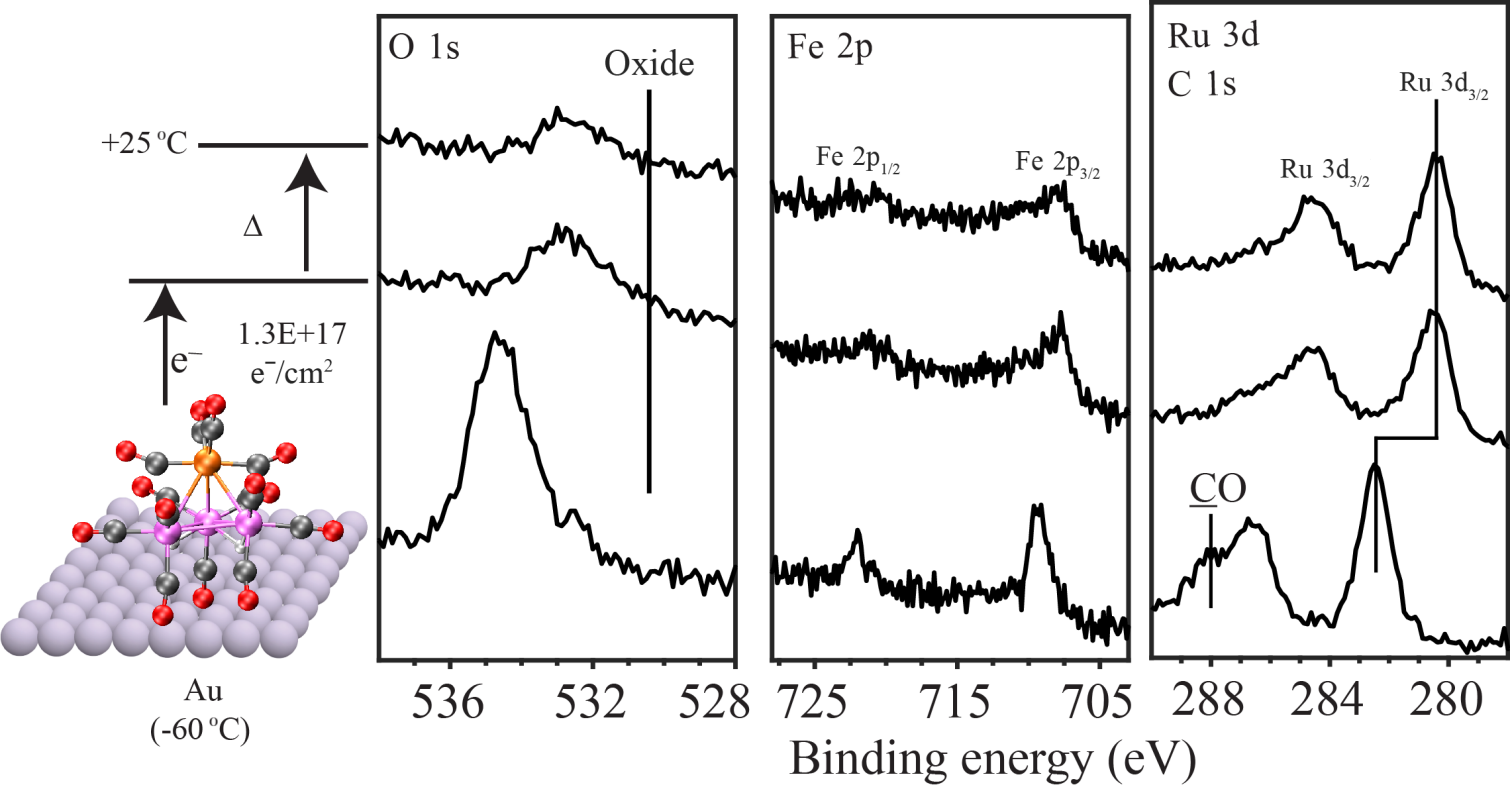
Changes in O 1s, Fe 2p and Ru 3d/C 1s XPS regions when an H_2_FeRu_3_(CO)_13_ film was exposed to electron dose of 1.3 × 10^17^ e^−^/cm^2^ (middle set of spectra) and then subsequently heated to room temperature (298 K) (uppermost set of spectra).

Figure 12 shows the evolution of the O 1s, Fe 2p and Ru 3d/C 1s XPS regions of a H_2_FeRu_3_(CO)_13_ film adsorbed at 213 K (bottom set of spectra), after exposure to an electron dose of 1.3 × 10^17^ e^−^/cm^2^ (middle spectra) and then subsequently annealed to RT (298 K) (topmost spectra). An initial electron dose of 1.3 × 10^17^ e^−^/cm^2^ was chosen because it closely corresponds to the minimum electron dose required to complete the initial stage of the reaction, in the regime where changes were observed in both the coverage of oxygen-containing species and the Ru peak position (see Figure 9 and Figure 10). In Figure 12 the changes in the O 1s, Fe 2p and Ru 3d/C 1s XPS regions for this electron dose (1.3 × 10^17^ e^−^/cm^2^) are seen to be similar to those shown in Figure 9, with the dominant effects being the loss of signal intensity in the O 1s region (≈70% of its initial value) and a decrease of ≈1.6 eV in the binding energy of the Ru atoms. Upon annealing this irradiated film to RT, the O 1s area decreased by a relative small amount (≈22% in intensity) but did not disappear; moreover, a slight shoulder was still visible on the higher binding energy side of the Ru 3d_3/2_ peak, supporting the idea of some residual CO ligands. No change was observed in the Ru 3d peak positions although there is a suggestion of slight broadening in the Fe 2p region.

**Discussion of surface science results:** Previous UHV-surface science studies on organometallic precursors have shown that the initial step in FEBID involves electron stimulated decomposition of the precursor accompanied by ligand desorption [43–44,46,49–50]. This causes the deposition of non-volatile metal-containing fragments that become incorporated into the deposit. In the present study, analysis of Figure 9 and Figure 10 reveals that this initial decomposition/deposition step is complete for electron doses on the order of 1.0 × 10^17^ e^−^/cm^2^ where the fraction of CO ligands present in the H_2_FeRu_3_(CO)_13_ film is reduced to about 30% of its initial value. This is evidenced by the decrease in intensity within the O 1s region and the evolution of CO detected by MS (Figure 11). Further, this electron stimulated H_2_FeRu_3_(CO)_13_ decomposition leads to decrease in the binding energy of the Fe 2p_3/2_ (709.1 to 708.5 eV) and Ru 3d_5/2_ (from 282.2 to 280.6 eV) peaks as the metal atoms are reduced; indeed, the Ru 3d_5/2_ binding energy of 280.6 eV after irradiation is close to the binding energy of Ru metal (280.1 eV), suggesting extensive CO desorption from the Ru atoms.

When comparing the extent of CO desorption from H_2_FeRu_3_(CO)_13_ [82] during the electron stimulated decomposition, it is apparent from Figure 10 that 65–70% of the CO groups present in the precursor desorb in the initial decomposition step. Based on the precursor’s stoichiometry we can therefore estimate that on average 8–9 of the 13 CO ligands present in H_2_FeRu_3_(CO)_13_ desorb during its electron stimulated decomposition. From the gas phase experiments described earlier we infer that the average CO loss per incident through DI is in the range from about 3–9. Similar analysis of the gas phase DEA data results in an average of 0.5 for the lower bound and 3 for the upper bound of CO loss through decomposition of H_2_FeRu_3_(CO)_13_ upon electron attachment. These two processes, along with neutral dissociation upon electron excitation, are expected to be responsible for the bulk of precursor decomposition in FEBID. Unfortunately, the extent of ND and the product formation in ND of FEBID precursors cannot be determined with currently available instruments, though the first steps in this direction have recently been taken [83]. However, as discussed in the gas phase section of this contribution we expect the extent of decomposition through neutral dissociation (ND) to be similar to that through dissociative electron attachment (DEA), rather than dissociative ionization (DI). From the comparison of the gas phase and surface data we thus expect DI to dominate the initial electron induced decomposition process observed for H_2_FeRu_3_(CO)_13_. However, it should be noted that the initial fragmentation of the precursor through DEA or DI could be followed by subsequent surface induced fragmentation, which cannot be excluded.

**Figure 13:**
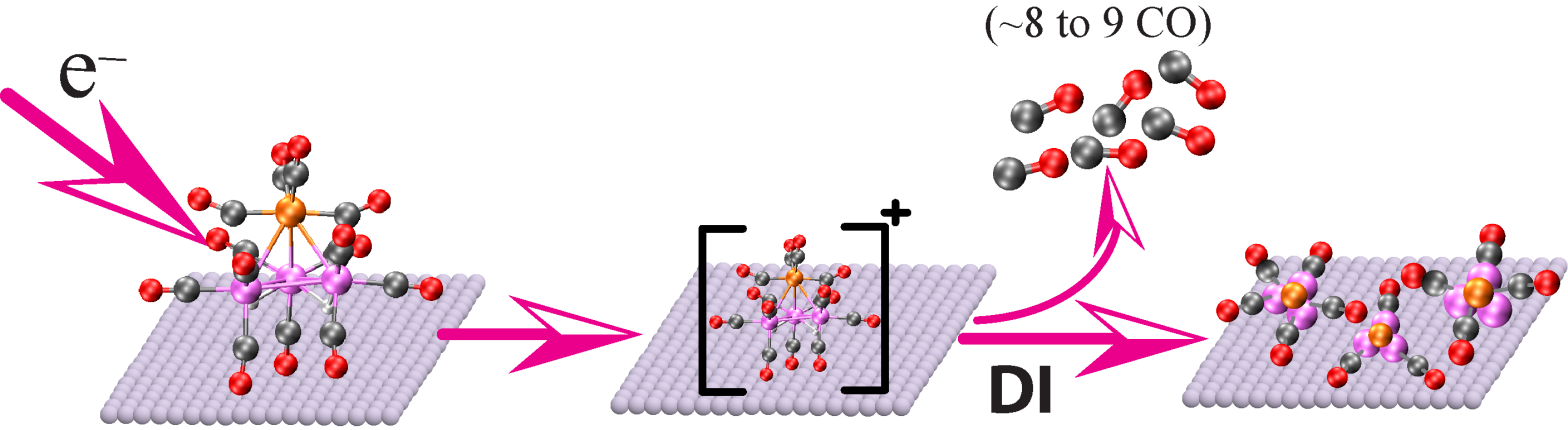
Initial decomposition/deposition of surface adsorbed H_2_FeRu_3_(CO)_13_ precursor, mediated by dissociative ionization. The mixture of partially decarbonylated intermediates shown on the right side represents the statistical nature of DI, each generated by the loss of a different number of CO groups from the parent molecule.

During precursor decomposition, CO desorption occurs in the absence of any CO decomposition, which previous studies have shown would lead to the appearance of a lower binding energy peak in the O 1s region (see Figure 9) as oxides are formed. Consequently, we can conclude that the parent H_2_FeRu_3_(CO)_13_ molecules are initially converted into partially decarbonylated, surface bound intermediates as shown in Figure 13. This schematic also highlights the fact that DI is an inherently statistical process. As a result, we do not expect that a single partially decarbonylated intermediate is formed, but rather a distribution of H_2_FeRu_3_(CO)*_x_* species with an average stoichiometry of H_2_FeRu_3_(CO)*_x_* (*x* = 4.5)_._ Unfortunately, XPS cannot detect hydrogen and H_2_ is a ubiquitous background gas in UHV chambers. As a result, the surface science studies do not provide any insights into the fate of the hydrogen atoms in the precursor.

**Fate of the partially decarbonylated intermediate:** In FEBID, deposition is typically conducted in an electron microscope at ambient temperatures under steady state conditions, where the partially decarbonylated intermediates formed by the decomposition of the precursors will be subjected either to the effects of further electron beam irradiation and/or undergo thermally stimulated reactions. In the UHV-surface science studies we can independently probe the fate of the partially decarbonylated intermediates towards further electron beam irradiation as well as their thermal stability at ambient temperatures.

**Stability towards further electron irradiation:** After an electron dose of ≈1 × 10^17^ e^−^/cm^2^ essentially all of the parent precursor molecules have undergone electron stimulated decomposition as evidenced by the absence of any Ru 3d_5/2_ peak at 282.2 eV associated with the parent compound as well as the invariance of the Ru 3d spectral envelope for electron doses in excess of ≈1 × 10^17^ e^−^/cm^2^. At this stage the precursor molecules have been converted into partially decarbonylated fragments as shown in Figure 13 with an average stoichiometry of H_2_FeRu_3_(CO)_4.5_. The XPS results shown in Figure 9 and Figure 10 for electron doses >≈1 × 10^17^ e^−^/cm^2^ therefore reflect the effect of further electron irradiation on these partially decarbonylated fragments. In typical FEBID experiments, surface bound intermediates will often be exposed to the effects of electron irradiation, as deposition occurs in the presences of a constant flux of electrons. Analysis of XPS data for electron doses >≈1 × 10^17^ e^−^/cm^2^ revealed no noticeable changes in the O 1s, Fe 2p and Ru 3d/C 1s regions. This suggests that the CO ligands of these partially decarbonylated intermediates are relatively stable towards electron irradiation. This is in contrast to previous studies of CO-containing FEBID precursors (e.g., W(CO)_6_ [43]) where the partially decarbonylated intermediates formed as the precursor decomposed were susceptible to electron induced decomposition of the CO ligands and the subsequent formation of metal oxides and graphitic carbon (M(CO)_y(ads)_ + e^−^ → MO(ads) + C(ads)). The reasons for the apparent stability of the CO ligands in the H_2_FeRu_3_(CO)*_x_* (*x* = 4,5) intermediates is unclear and somewhat surprising. In the context of its influence on the metal content in FEBID structures, however, the apparent persistence of these CO ligands under the influence of electron irradiation is no different to the effect of CO decomposition as both routes will cause the associated carbon and oxygen atoms to remain in the deposit as it grows.

**Thermal stability:** In contrast to the low temperature (213 K) UHV surface science studies where experiments in this study are principally conducted, FEBID occurs at ambient temperatures (≈298 K). In FEBID, although the initial step must involve electron mediated decomposition/deposition of the precursor, the surface bound intermediates formed as a result of precursor decomposition could subsequently react thermally. To assess the potential for this to occur it is necessary to assess the thermal stability of the partially decarbonylated intermediates generated from H_2_FeRu_3_(CO)_13_. Experimentally, we accomplish this in Figure 12 using XPS by first exposing H_2_FeRu_3_(CO)_13_ films to an electron dose sufficient to create the partially decarbonylated intermediates, and then annealing these species to RT. Analysis of Figure 12 reveals that there is loss of some of the O 1s intensity (O 1s area reduced by ≈22%), but most of the CO groups in the H_2_FeRu_3_(CO)*_x_* (*x* = 4,5) intermediates still remain with average stoichiometry of H_2_FeRu_3_(CO)*_x_* (*x* = 3,4).

FEBID nanostructures made from H_2_FeRu_3_(CO)_13_ under steady state conditions have metal contents <26 atom % as discussed vide infra. From the surface science results discussed so far, it is clear that the partially decarbonylated intermediates generated from H_2_FeRu_3_(CO)_13_ (H_2_FeRu_3_(CO)*_x_*) will not change significantly in terms of their chemical composition, regardless of whether they are subject to the effects of further electron beam irradiation (Figure 10) or thermal processing (Figure 12). Consequently, most of the associated carbon and oxygen atoms residual in the partially decarbonylated intermediates will be incorporated into the growing deposits. Indeed, the overall sequence of elementary reaction steps H_2_FeRu_3_(CO)_13_ precursor molecules will experience in FEBID can be represented as:

H_2_FeRu_3_(CO)_13_ (physisorbed) + e^−^ → H_2_FeRu_3_(CO)*_x_* (*x* = 4,5) (chemisorbed) + 8.5 (CO)(g)↑

H_2_FeRu_3_(CO)*_x_* (*x* = 4,5) (chemisorbed) + e^−^/Δ → most CO ligands are retained.

**Figure 14:**
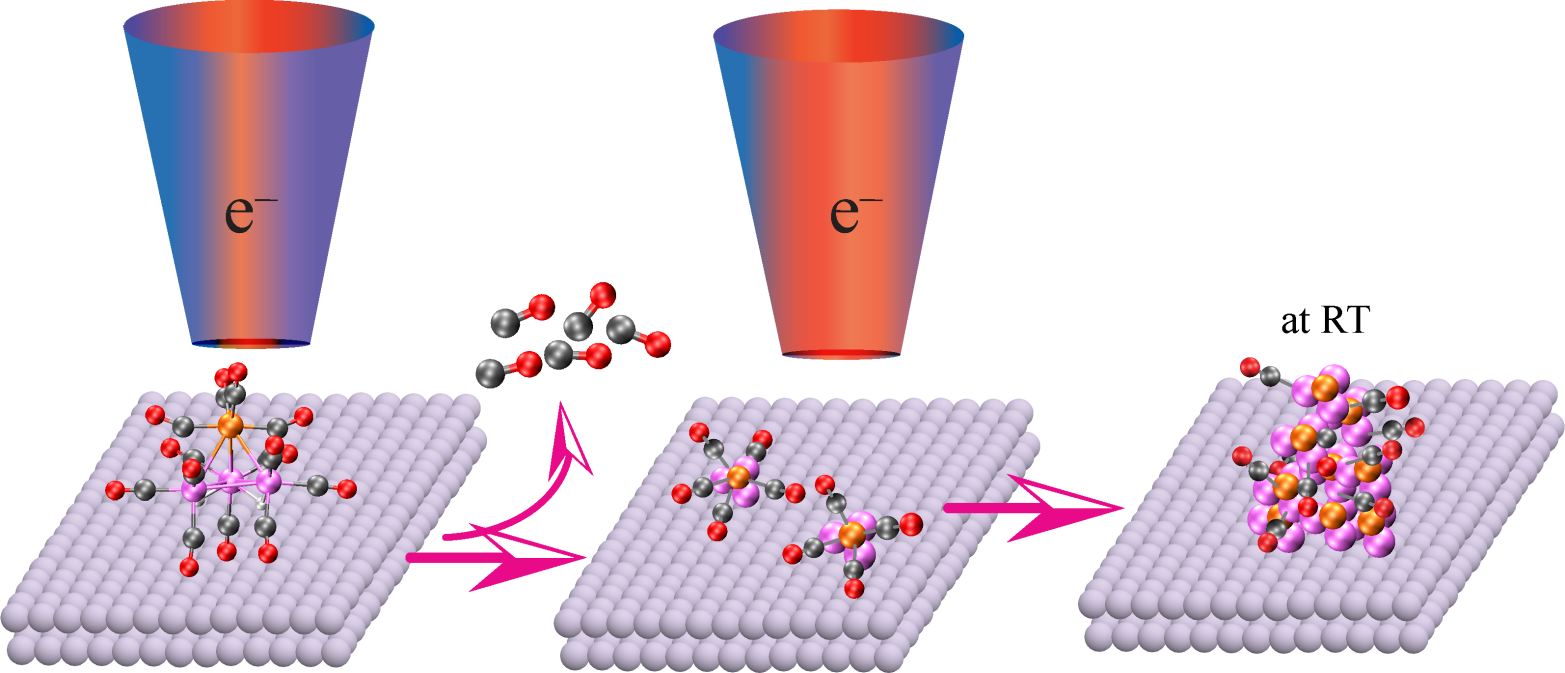
Schematic showing the incorporation of partially decarbonylated intermediate of H_2_FeRu_3_(CO)_13_ into the deposit in FEBID.

This overall process is shown schematically in Figure 14, where the partially decarbonylated intermediates are incorporated into the FEBID nanostructure. From the stoichiometry of the film formed after irradiation and annealing of surface adsorbed H_2_FeRu_3_(CO)_13_ film, one can estimate the metal content and the estimated value is <31%. These values match very well with the composition obtained in the current FEBID experiments with H_2_FeRu_3_(CO)_13_. As is discussed in the next section the metal content varies in the range 21 to 26 atom % with optimized deposition parameters in FEBID of H_2_FeRu_3_(CO)_13_.

Related experiments on the electron induced decomposition of surface adsorbed HFeCo_3_(CO)_12_ indicate a similar behavior with regards to the initial electron induced decomposition. However, for HFeCo_3_(CO)_12_, thermal CO desorption from the partially decarbonylated intermediates is essentially complete at room temperature, in contrast to the behavior of H_2_FeRu_3_(CO)_13_ [80].

### Focused electron beam induced deposition of H_2_FeRu_3_(CO)_13_

Heteronuclear precursors are attractive for FEBID of alloys and intermetallic compounds, in particular, if the deposit metal composition is in accordance with the stoichiometric proportions of the metal species in the precursor and if the precursor leads to deposits which are, ideally, fully metallic.

Prominent applications fields are nanomagnetism, in particular regarding the advent of 3D nanomagnetic structures [84–85], or superconducting nanostructures for studying finite-size and (quantum) phase slip effects or the direct writing of device structures based on tunneling or Andreev reflection [86–88].

In recent work we have shown that HFeCo_3_(CO)_12_ is a rather ideal precursor with regard to preserving the metal composition in FEBID and leading to deposits with high metal content (typically above 80 atom %) for a wide range of deposition conditions, in particular those which are suitable for high-resolution structure formation [66].

In the following we present new results concerning the performance of H_2_FeRu_3_(CO)_13_ as precursor in FEBID and compare this to our previous results on HFeCo_3_(CO)_12_.

**Dependence of gas flux on deposit composition for H_2_FeRu_3_(CO)_13_:** In a first series of experiments we followed the evolution of the deposits’ composition as we increased the precursor temperature in several discrete steps under otherwise fixed deposition conditions of 5 keV beam energy and 1.6 nA beam current. The pitch was set to 20 nm in both, *x* and *y* direction, and the dwell time was fixed to 1 µs. In Table 1 we present an overview of the composition evolution for different precursor temperatures in the first-time heating process after the initial precursor loading, and in a follow-up experiment, when the precursor had already reached the highest temperature of 338 K accessible in our setup. In these experiments we also varied the distance of the gas injection capillary to the substrate surface and the field of view.

**Table 1:** Determination of the optimal gas injection system (GIS) temperature. For the confirmation of the precursor’s stability under heating, the experiments were repeated after the precursor had once been heated to 338 K. The deposits were written with 5 keV, 1.6 nA, 20 nm pitch and a dwell time of 1 µs. The chemical deposition was determined via EDX. Signal contributions from the substrate, Si/Si_3_N_4_, as visible by the spectral contributions of Si, were taken out from the EDX quantification. LDFOV: lateral distance to the center of the field of view. DS: vertical distance to the substrate.

Temperature (K)	GIS		Chemical composition (atom %)			
	LDFOV (µm)	DS (µm)	C	O	Fe	Ru

1st experiment: first heating of precursor after initial precursor loading						
313	90	100	23.4	75.5	0	1.1
315	90	100	30.3	67.8	0.3	1.6
323	90	100	35.1	62.3	0.4	2.2
338	65	110	40.9	51.4	1.6	6.1
2nd experiment: heating after initial precursor conditioning up to 65 °C						
298	103	85	24.7	75.0	0.3	0.0
303	100	90	17.8	82.0	0.2	0.0
313	104	85	27.0	72.2	0.8	0.0
323	101	90	27.0	69.6	1.1	2.3
333	100	93	43.8	42.7	3.0	10.5
338	100	80	44.4	34.4	4.7	16.5

From the data presented in Table 1 it is apparent that the highest metal content is obtained for the highest precursor flux, i.e., at 338 K. This is in clear correspondence to HFeCo_3_(CO)_12_ for which sufficient precursor flux can also only be obtained above 333 K. However, already at this stage a clear difference to HFeCo_3_(CO)_12_ is noticeable. Even at the highest flux the overall metal content of Fe–Ru does only reach 21 atom %, which is far below the typical value of 80 atom % of Fe–Co under the beam conditions employed here.

**FEBID growth optimization:** In a next step the FEBID process was optimized with regard to the metal composition by varying the beam current, *x* and *y* pitches and the dwell time. This was done with support from a semi-automatic non-linear optimization routine using a genetic algorithm (GA), as described in [89]. As a general trend, from the GA we find short dwell times and rather large pitches to result in the highest conductance for the deposits. A more detailed account is given in Table 2, which summarizes the results for a subset of the growth conditions investigated in this work.

**Table 2:** Overview of sample composition and room-temperature resistivity employing a set of standard writing parameters, as well as optimized parameters, as indicated. For the EDX experiments dedicated reference structures of sufficient thickness were fabricated. For these we employed the same deposition parameters as for the structures used for the transport measurements.

Current (nA)	Dwell time (µs)	*x* pitch (nm)	*y* pitch (nm)	Chemical composition				resistivity (Ω·cm)
				C	O	Fe	Ru	

standard writing parameters								
1.6	0.2	20	20	51.7	32.3	3.6	12.4	1.8 × 10^5^
1.6	1	20	20	49.2	41.6	1.9	7.3	9.2 × 10^3^
1.6	100	20	20	47.9	41.0	2.1	9.0	650
1.6	500	20	20	53.0	37.5	2.5	7.0	332
6.3	0.2	20	20	52.8	28.4	5.1	13.7	135
6.3	1	20	20	48.1	29.8	5.4	16.7	45.6
6.3	100	20	20	49.6	25.8	5.2	19.4	57.3
6.3	500	20	20	49.5	24.2	5.1	21.2	70.7
optimized writing parameters								
1.6	0.3	80	74	40.5	36.5	4.7	18.3	2.55
1.6	3.7	80	74	42.2	33.9	5.1	18.8	4.38
6.3	0.4	29	31	37.8	40.8	5.0	16.4	2.46
6.3	1.1	29	31	40.5	38.7	4.3	16.5	3.66

Three main results can be stated using H_2_FeRu_3_(CO)_13_: (i) The largest metal content for carefully optimized deposits does not reach beyond 26 atom %, which is significantly higher than the metal content obtained for other Ru-based precursors (≈10%) [61] but also very low when compared to HFeCo_3_(CO)_12_ (>80 atom %) [66]. (ii) The resistivity, however, is not lowest for the highest metal content but reaches its minimum for [Fe–Ru] ≈21 atom %, namely about 2.5 Ω·cm. (iii) There is a significant variation in the Ru-to-Fe ratio, reaching from 2.7 to 4.3. This is again in stark contrast to our observations for deposits obtained from HFeCo_3_(CO)_12_ for which the lowest resistivities, of below 100 µΩ·cm, nicely correlate with the largest metal content. Moreover, the large resistivity values of the Fe–Ru deposits clearly indicate that these are not metallic but are in the thermally-assisted tunneling regime; see [90] for a discussion of the electronic transport regimes of nanogranular FEBID materials. This is also clearly seen in the temperature-dependent conductivity, which we discuss in the last part of this section. We conclude this paragraph by commenting on the observed strong variability of the Ru-to-Fe ratio. Here significant deviations from the 3-to-1 ratio expected from the precursor composition are observed. In most instances the ratio exceeds 3, which indicates a partial loss of Fe during the dissociation and fragment desorption processes at work in FEBID for this precursor. With a view to the results from the gas phase experiments, this may be a consequence of the significant apex loss as neutral Fe(CO)_4_ in H_2_FeRu_3_(CO)_13_, as apparent in the current gas phase studies while the loss of anionic Fe(CO)_4_^−^ is the predominant apex loss channel in HFeCo_3_(CO)_12_. Furthermore metal–metal bond rupture, to a large extent through apex loss, is a pre-requisite for further fragmentation of H_2_FeRu_3_(CO)_13_ in the gas phase, but is only a parallel process to sequential CO loss in HFeCo_3_(CO)_12_.

**Figure 15:**
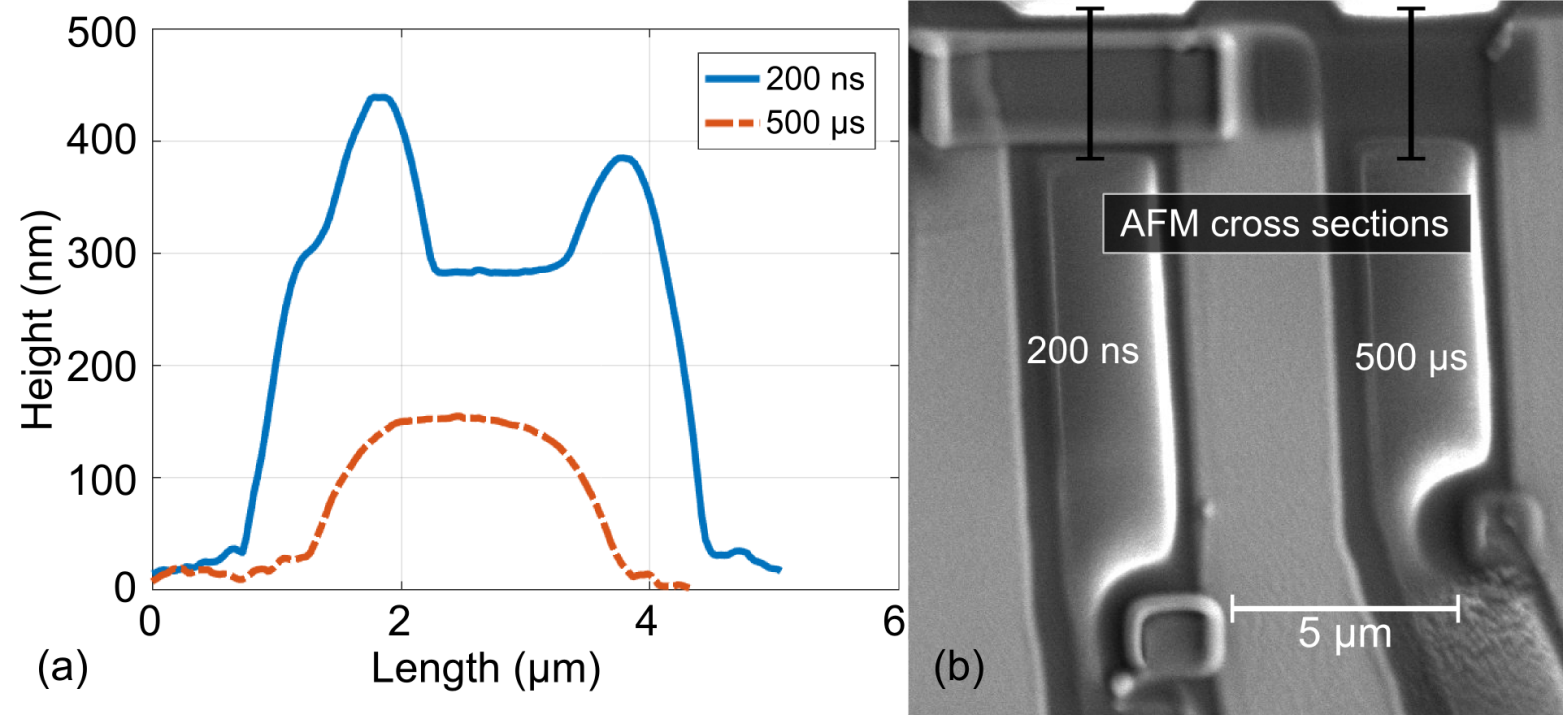
(a) AFM cross sections of deposits shown in the SEM micrograph (b) at the positions indicated by the black bars. The deposits were written with 5 keV, 6.3 nA and 20 nm pitch. The lateral shape of the patterns was set to be rectangular of size 6 × 2 µm^2^. For short dwell time (200 ns) the deposit’s dimensions exceed the pattern size by about a factor of two due to co-deposit formation.

**Deposit morphology and co-deposit formation:** Utilizing the deposition parameters from the optimization process described in the previous paragraph, we have fabricated rectangular reference structures for getting insight into the morphology of the deposits and the manifestation of co-deposits. In Figure 15a we show AFM cross section profiles of two deposits defined as a rectangle of size 6 × 2 µm^2^ for two different dwell times. The beam parameters were set to 5 keV and 6.3 nA.

Considering the extremely pronounced edge bulging for the short dwell time of 200 ns and the rounded shape for longer dwell times (500 µs), we attribute these morphological changes to a transition from a reaction-rate limited (RRL) to a mass-transport limited (MTL) regime with increasing dwell time. This is analogous to our previous results for the precursor W(CO)_6_ described in [91]. For short dwell times the dish-shape is particularly strongly pronounced and does in fact lead to a lateral inflation of the targeted size by a factor of two. This indicates that the morphological evolution is not only the consequence of the RRL growth regime but also of a strongly pronounced co-deposit formation. This is again in stark contrast to our observations regarding the deposit shape of Fe–Co structures obtained from HFeCo_3_(CO)_12_. Here we find only very small co-deposit contributions and nice shape fidelity under growth conditions optimized for both high metal content and high-resolution writing.

**Figure 16:**
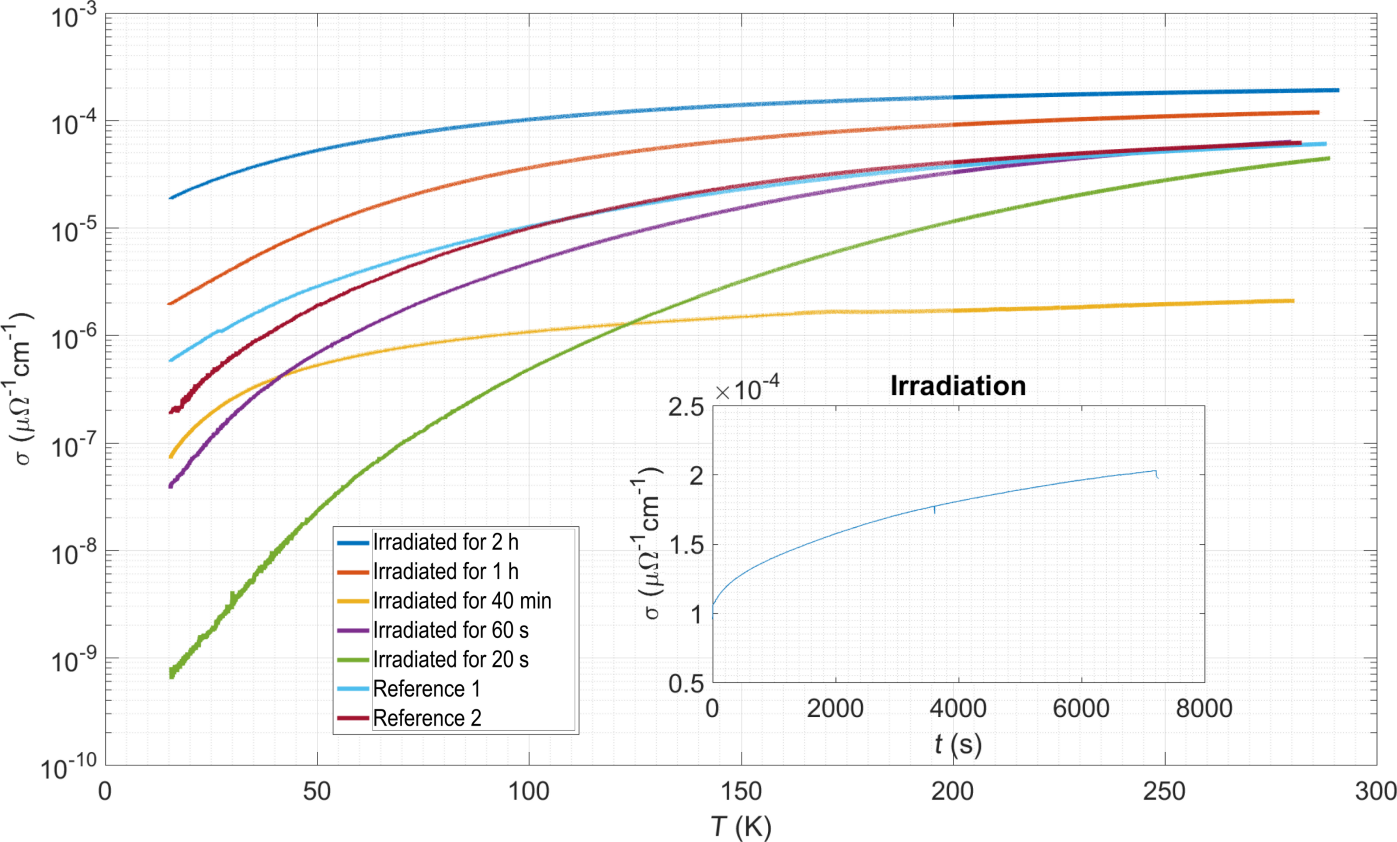
Transport measurements of as-grown Fe–Ru deposit and deposits grown under identical conditions after being subjected to different electron post-growth treatments, as indicated. The samples irradiated for 1 h and 2 h show the highest room-temperature conductivities, associated with the smallest reduction in conductivity as the temperature is lowered. Inset: Time-dependent evolution of the in situ conductivity as a sample is electron irradiated in the electron microscope after growth.

**Electronic transport regime of Fe–Ru FEBID structures:** In the last part of this section we briefly summarize our observations regarding the temperature-dependent conductivity of Fe-Ru FEBID nanostripes optimized for high metal content and being subject to a sequence of electron irradiation steps. For nanogranular Pt FEBID structures, post-growth irradiation has been shown to be very efficient in increasing the conductivity by up to four orders of magnitude, even up to the transition from a thermally assisted tunneling regime to a metallic transport regime [92–93]. In Figure 16 we show the temperature-dependent conductivity of Fe–Ru nanostripes prepared at 5 keV and 6.3 nA beam energy and current, respectively. The dwell time was set to 400 ns at a symmetric pitch of 80 nm.

As is apparent from the inset of Figure 16, the effect of post-growth electron irradiation on the conductivity of Fe–Ru deposits is noticeable, but even for extended irradiation times (2 h) the increase is just below a factor of two. The main plot in Figure 16 gives an overview of the overall temperature-dependence of Fe–Ru nanostripes written under identical conditions but being subject to irradiation times of 20 s up to 2 h. A comparison of any of the two as-grown reference structures with the nanostripe irradiated for only 20 s indicates that the overall variability in the activation temperature of these semiconducting/insulating deposits is rather large. The observation of a strongly activated transport, dominated by tunneling, is again in clear contrast to our observations on Fe–Co from HFeCo_3_(CO)_12_, for which we routinely observe metallic behavior, i.e., increasing conductivity as the temperature is lowered, at room-temperature resistivity levels which are at least 4 orders of magnitude lower than those observed for optimized Fe–Ru.

## Conclusion

A comprehensive investigation of electron interactions with the FEBID precursor H_2_FeRu_3_(CO)_13_ was conducted under different experimental conditions, using gas phase and UHV surface-based studies as well as focused electron beam induced deposition (FEBID).

In the current FEBID of H_2_FeRu_3_(CO)_13_, considerable effort was given to optimize the resulting metal content of the deposit, however, only a maximum metal content of about 26 atom % was achieved. Moreover, the metal content was generally found to be lower than 26 atom %. This is a quite poor performance compared to the structurally similar precursor HFeCo_3_(CO)_12_, where stoichiometric metal content of 80 atom % was routinely achieved [66]. EDX analysis of the deposits also produced a Ru/Fe ratio that was higher (average Ru/Fe ≈3.5:1) than the stoichiometric ratio of 3:1 in the intact precursor.

In gas phase electron interaction with H_2_FeRu_3_(CO)_13_ all CO loss through DEA is preceded by metal–metal bond ruptures, with a significant apex loss in the form of neutral Fe(CO)_4_. In DI, metal–metal bond rupture is also significant and is observed for more than 50% of all dissociation events. The average CO loss from H_2_FeRu_3_(CO)_13_ through DI is found to be between 3 and 9 per incident and in DEA these numbers are about 0.5 to 3. In the surface experiments, decomposition of the parent molecules leads to an average loss of 8–9 CO groups and the formation of partially decarbonylated intermediates. The partially decarbonylated intermediates (average stoichiometry H_2_FeRu_3_(CO)_4.5_) formed as a result of the precursor’s decomposition are relatively unaffected by further electron irradiation or thermal annealing to room temperature (where typical FEBID experiments are conducted).

Comparing the extent of the CO loss through DEA and DI in the gas phase experiments with the observed CO loss in the surface experiments indicates that DI, rather than DEA is the dominating process for this precursor. However, we note that ND is not accounted for in current gas phase experiments and this process may be significant. Fragmentation through ND is expected to be more similar to DEA than DI, and should be manifested in a similar extent of CO loss. The fact that no desorption of metal containing fragments is observed in the surface experiments despite the fact that these dominate the gas phase fragmentation observed may readily be explained by the low substrate temperature in the surface experiments (213 K).

The main difference in the gas phase fragmentation of H_2_FeRu_3_(CO)_13_ as compared to HFeCo_3_(CO)_12_ is the significantly more pronounced metal–metal bond ruptures observed for H_2_FeRu_3_(CO)_13_. Especially the apparently significant apex loss through the formation of neutral Fe(CO)_4_ is of interest as this fragment could desorb at room temperature during FEBID. Related surface studies on HFeCo_3_(CO)_12_ reveal that the initial electron induced decomposition of this precursor is similar to that of H_2_FeRu_3_(CO)_13_. However, post exposure annealing to room temperature apparently allows for more efficient removal of the remaining CO ligands from HFeCo_3_(CO)_12_ than is the case for H_2_FeRu_3_(CO)_13_ [80].

With the observations from the surface science and gas phase studies we can attribute the low metal content achieved with H_2_FeRu_3_(CO)_13_ predominantly to the persistence of the CO ligands that remain after the initial electron induced decomposition. Id est, for H_2_FeRu_3_(CO)_13_ these are not effectively removed through annealing to room temperature in the surface experiments. Consequently, the associated carbon and oxygen atoms are likely to be incorporated in FEBID nanostructures, decreasing the metal content. Moreover, the final metal content of the deposits observed for H_2_FeRu_3_(CO)_13_ in the surface experiments is very similar to that achieved in FEBID under optimal conditions, i.e., 31 vs 26 atom %. The vapor pressure of H_2_FeRu_3_(CO)_13_ is also very low in FEBID experiments as compared to the background gases. This will increase the relative importance of deposition from background gases, which likely explains the observation that the metal content was often significantly less than the maximum value of 26 atom % observed. Gas phase studies suggest that the slight increase in the Ru/Fe ratio of deposits from that of the molecular stoichiometry, could be a reflection of the desorption of neutral, iron containing ligands, predominantly Fe(CO)_4_.

## Experimental

**Preparation of the heterometallic carbonyl precursors** was conducted through modified procedures described in references [94–95]. Our modified procedure for HFeCo_3_(CO)_12_ has been described in the literature [66], while the procedure for the synthesis of H_2_FeRu_3_(CO)_13_ was conducted as follows (CIF file with refined data for H_2_FeRu_3_(CO)_13_ can be provided upon request).

All handling and synthesis procedures were carried out under inert atmosphere using Schlenk and glove box techniques to prevent oxidation. Fe(CO)_5_, Ru_3_(CO)_12_, H_3_PO_4_, benzene, hexane, 1,4-dioxane, and acetone were purchased from Sigma Aldrich. All solvents were deoxygenated before use. Na_2_[Fe(CO)_4_] is formed by reduction of Fe(CO)_5_ in THF using a sodium/benzophenone mixture as described in literature [96]. The solid product is filtered, washed with hexane, dried under vacuum and stored in a glovebox. In a typical synthesis, 200 mg Ru_3_(CO)_12_ in 70 mL THF was added dropwise to a refluxing solution of 140 mg Na_2_[Fe(CO)_4_] in 80 mL THF. The colorless solution turned red upon the first addition of Ru_3_(CO)_12_ and was refluxed for additional 75 min after complete mixing of the compounds. The solvent was subsequently removed from the solution under reduced pressure. Hexane was added to the residue and further addition of 30 mL 20% H_3_PO_4_ led to a coloration of the hexane phase due to phase transfer. The organic phase was pipetted in another flask containing anhydrous MgSO_4_, filtered and concentrated under reduced pressure. The concentrate was chromatographically purified under argon atmosphere using silica gel (column length 30 cm; Ø 3 cm) and hexane as eluent with the column length being sufficient to clearly separate the different fractions containing other metal carbonyls. Three fractions of distinct color (yellow, green and purple) were eluted with hexane and discarded. The red product was finally stripped from the column using benzene and the solvent was removed. The crude product was crystallized from hexane to obtain sheet-like crystals of H_2_FeRu_3_(CO)_13_, which were dried under reduced pressure. ^1^H NMR identified a small concentration of impurity hexanes with very low concentration in a saturated solution. These are however removed under reduced pressure. Crystals were checked by single crystal XRD (with *R* = 2.8%) without any solvent molecules incorporated in the crystals. Unit cell parameters were determined for different crystals and gave the same results. IR spectra are recorded, and were found to be in accordance with literature data. The thermal stability of H_2_FeRu_3_(CO)_13_ was investigated by thermogravimety with a heating rate of 5 K·min^−1^. The onset of thermal decomposition for the precursor is at ≈390 K. Since the stability at elevated temperatures is important for the evaporation of the precursor, isotherms at different temperatures under nitrogen were recorded. The most important one for the evaporation procedure showed a mass loss of <1.5% during heating for 4 h at 343 K.

**Gas phase experiments** were conducted in a crossed electron/molecular beam instrument under single collision condition. A full description of the experimental setup can be found in [97] and thus only a brief description is given here. An electron beam with energy resolution of ≈110 meV was generated using a trochoidal electron monochromator and crossed with an effusive beam of H_2_FeRu_3_(CO)_13_ produced by subliming the precursor molecule into the collision chamber through a capillary tube. The flow of the molecular beam can be precisely controlled with a leak valve. Prior to the measurement, the background pressure of the collision chamber was ≈6 × 10^−8^ mbar. With heating of the inlet system to 338–343 K, the precursor pressure increased to 2–4 × 10^−7^ mbar. In order to avoid charging of the electrical lense components of the monochromator due to condensation on its surfaces, the monochromator was maintained at a temperature of about 393 K. The electron energy scale was calibrated based on the SF_6_^−^/SF_6_ formation at 0 eV and the energy resolution of the electron beam was estimated from the FWHM of the SF_6_^−^/SF_6_ signal at 0 eV. Both positive and negative ions formed by the interaction of electrons with H_2_FeRu_3_(CO)_13_ were analyzed and detected using a quadrupole mass spectrometer (Hiden EPIC1000).

Compared to HFeCo_3_(CO)_12_ the handling of H_2_FeRu_3_(CO)_13_ was more demanding. Maintaining sufficient pressure for acceptable signal to noise ratios and acquisition times required temperatures above 338 K, preferably higher, while heating above 348–353 K caused decomposition of H_2_FeRu_3_(CO)_13_ (identified by decrease of relevant fragment ion signals). For HFeCo_3_(CO)_12_, on the other hand, it was easy to maintain sufficient working pressure and no change in the behavior of this compound was observed, even at temperatures as high as 358 K.

**The quantum chemical calculations** of the thermochemical thresholds for the negative ion fragment formation were performed using the ORCA computational chemistry software [98]. In most of the calculations we tried multiple ways of removing CO ligands and other respective fragments, then for each possibility we optimized the structures at the BP86 [99–100] /def2-TZVP [76] level of theory to the most probable minimum energy structure. However, it was not practical to consider all possible structures. After geometry optimizations, the single point energies were calculated with the hybrid DFT functional PBE0 [75–76] and the basis set ma-def2-TZVP [76–77]. The energetics and threshold calculations reported in the current work are the best optimized values obtained in our quantum chemical calculations. Since the H_2_FeRu_3_(CO)_13_ precursor molecule was heated during the experiments, we included the thermal energy of the neutral molecule in the calculations (at 343 K the calculated thermal energy of H_2_FeRu_3_(CO)_13_ is 1.16 eV). We note, that this is the most probable internal energy and does not account for the actual Maxwell–Boltzmann distribution of internal energies at the current temperature. The molecular orbitals and spin density of H_2_FeRu_3_(CO)_13_ were plotted using VMD [101].

**The surface science experiments** were performed in a UHV-chamber equipped with XPS and MS (more details can be found in [44,102]). A gold substrate was used because it is chemically inert and because there are no Au XPS or AES peaks which overlap with any Ru 3d/C 1s, Fe 2p or O 1s XPS peaks. Prior to each experiment, the Au surface was cleaned by sputtering with 4 keV Ar^+^ ions. To create H_2_FeRu_3_(CO)_13_ films, H_2_FeRu_3_(CO)_13_ was sublimed into the UHV-chamber from a glass finger through heating to 338–343 K. The heating increased the chamber pressure from ≈9 × 10^−9^ mbar to ≈4 × 10^−7^ mbar. H_2_FeRu_3_(CO)_13_ was deposited onto a cooled (153 K) Au substrate. To get sufficiently thick films, it was necessary to dose the precursor continuously for 4 h. The thickness of the film was determined from the attenuation of the Au 4f XPS signal, using an inelastic mean free path of 2 nm for the Au 4f photoelectrons [103]. Typical H_2_FeRu_3_(CO)_13_ film thickness ranged from 1.1 to 1.4 nm. Based on the effective size of the H_2_FeRu_3_(CO)_13_ molecule (estimated from the computed structure of H_2_FeRu_3_(CO)_13_) this film thickness corresponds to 1 to 2 monolayers. The composition of the film was determined from analysis of the Ru 3d/C 1s, Fe 2p and O 1s XPS transitions.

Following deposition, the substrate temperature was increased from 153 K to 213 K to ensure that any co-adsorbed water had desorbed prior to electron irradiation. A commercial flood gun (Spec FG 15/40) was used to irradiate the adsorbed film of H_2_FeRu_3_(CO)_13_. For all surface science experiments, we used an electron energy of 500 eV. This value corresponds to the sum of the flood gun's electron energy and a positive bias of +20 eV applied to prevent the escape of secondary electrons generated in the surface by the impact of primary electrons. Electron flux was measured based on the target current, which was held constant during a particular experiment by adjusting the electron current as needed. Changes in the film’s composition and bonding as a result of electron irradiation were determined by measuring the Ru 3d/C 1s, Fe 2p and O 1s transitions in terms of electron dose (electron dose = target current × exposure time).

All XPS data were recorded with a PHI 5400 XPS using Mg Kα X-rays (*h*ν = 1253.6 eV). The measured spectra were de-convoluted using commercial software CASA XPS. The Ru 3d/C 1s, Fe 2p and O 1s regions were calibrated by aligning the measured Au 4f_7/2_ substrate XPS peak to 84 eV. Gas phase species which desorbed from the surface as a result of electron irradiation were monitored using a quadrupole mass spectrometer (Stanford research system, 0 to 200 amu).

**The FEBID experiments** were performed in a dual beam microscope (FEI Nova NanoLab 600) equipped with a Schottky electron emitter. The base pressure of the system was 4 × 10^−6^ mbar. An Omniprobe gas injector with inner diameter of the capillary of 0.5 mm was used. The distances of the capillary to the substrate surface and the center of the field of view was 100 µm (vertically) and 90 to 100 µm (laterally), respectively. The precursor HFeCo_3_(CO)_12_ was heated to 337 K leading to an increase of the chamber pressure by about 1 × 10^−7^ mbar. The precursor H_2_FeRu_3_(CO)_13_ was heated in several steps up to 338 K leading to a hardly detectable increase of the chamber pressure. The deposition experiments were done on p-doped Si(100) substrates with native oxide, thermally grown SiO_2_ (200 nm) or Si_3_N_4_ (100 nm). We did not observe an appreciable influence of the different substrate materials on the results of the deposition experiments. The substrate temperature was 296 K for all deposition experiments. After some preliminary experiments we set the beam energy to 5 keV for the deposition experiments with H_2_FeRu_3_(CO)_13_, as we observed a strong reduction in both deposition yield and metal content at higher beam energies. For HFeCo_3_(CO)_12_ we have shown in previous work that high metal contents can be obtained for a wide range of beam energies and currents [66].

Energy-dispersive X-ray analysis (EDX) experiments were performed in situ directly after growth at 5 keV beam energy and 1 nA beam current.

Transport measurements were performed in the temperature range from 2 to 300 K under fixed bias voltage conditions in two- or four-probe configuration, employing Cr/Au electrode structures prepared by dc magnetron sputtering and standard UV lithography.

Atomic force microscopy (Nanosurf EasyScan 2) in dynamic, non-contact mode was done on selected samples.

## Supporting Information

File 1 Additional data.
