# Supplementary material for: Electron interactions with the heteronuclear carbonyl precursor H2FeRu3(CO)13 and comparison with HFeCo3(CO)12: from fundamental gas phase and surface science studies to focused electron beam induced deposition
Source: Beilstein J Nanotechnol. 2018 Feb 14;9:555–79. doi: 10.3762/bjnano.9.53 (PMC5827713; doi:10.3762/bjnano.9.53)
Supplement: File 1 — Additional data. [file Beilstein_J_Nanotechnol-09-555-s001.pdf]

Supporting Information for

Electron Interactions with the heteronuclear carbonyl precursor ($H_2FeRu_3(CO)_{13}$) and comparison with $HFeCo_3(CO)_{12}$: from fundamental gas phase and surface science studies to focused electron beam induced deposition

Ragesh Kumar T P[1], Paul Weirich[2], Lukas Hrachowina[3], Marc Hanefeld[2], Ragnar Bjornsson[1], Helgi Rafn Hrodmarsson[1], Sven Barth[3], D. Howard Fairbrother[4], Michael Huth[2], and Oddur Ingólfsson*[1]

Address

[1]Science Institute and Department of Chemistry, University of Iceland, Reykjavík, Iceland

[2]Physikalisches Institut, Max-von-Laue-Str. 1, Goethe-Universität, 60438 Frankfurt am Main, Germany

[3]Institute of Materials Chemistry, TU Wien, 1060 Vienna, Austria

[4]Department of Chemistry, Johns Hopkins University, Baltimore, Maryland, USA

Email: odduring@hi.is

* Oddur Ingólfsson

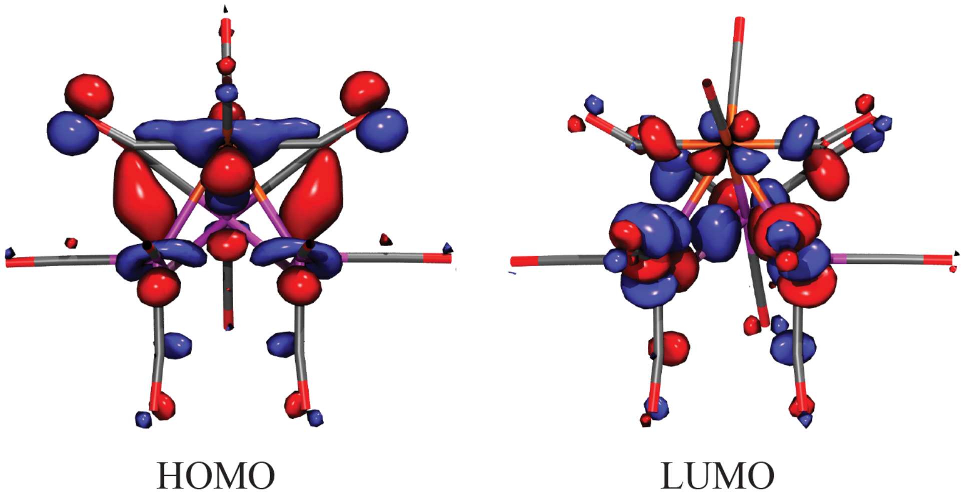

Figure S1: The highest occupied molecular orbital (HOMO) and lowest unoccupied molecular orbital (LUMO) of $H_2FeRu_3(CO)_{13}$.

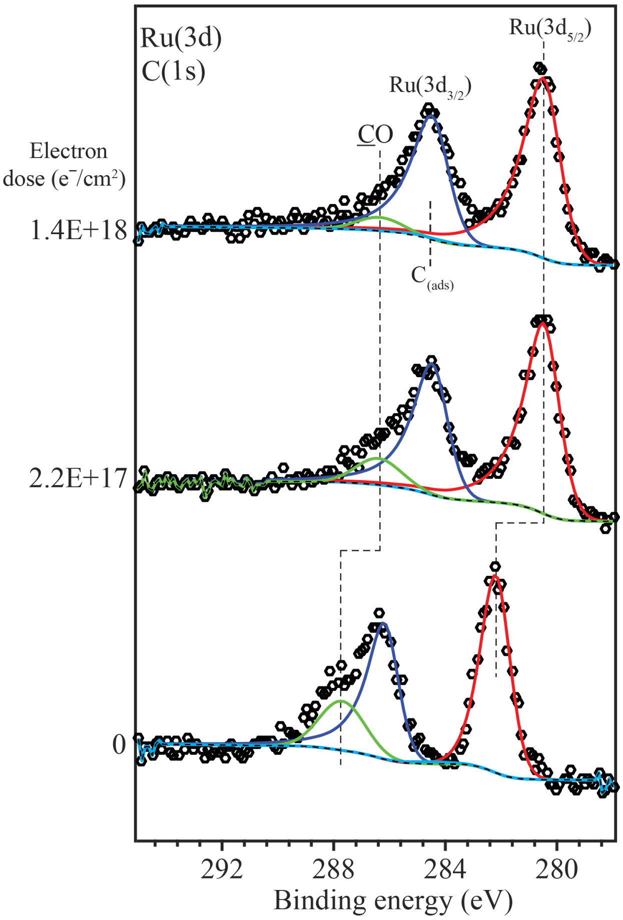

Figure S2: Fitting of Ru(3d)/C(1s) XPS region prior to electron irradiation and with electron dose of $2.2x10^{17}$ e^-/cm^2 and $1.4x10^{18}$ e^-/cm^2, XPS regions are fitted using CASA XPS program.

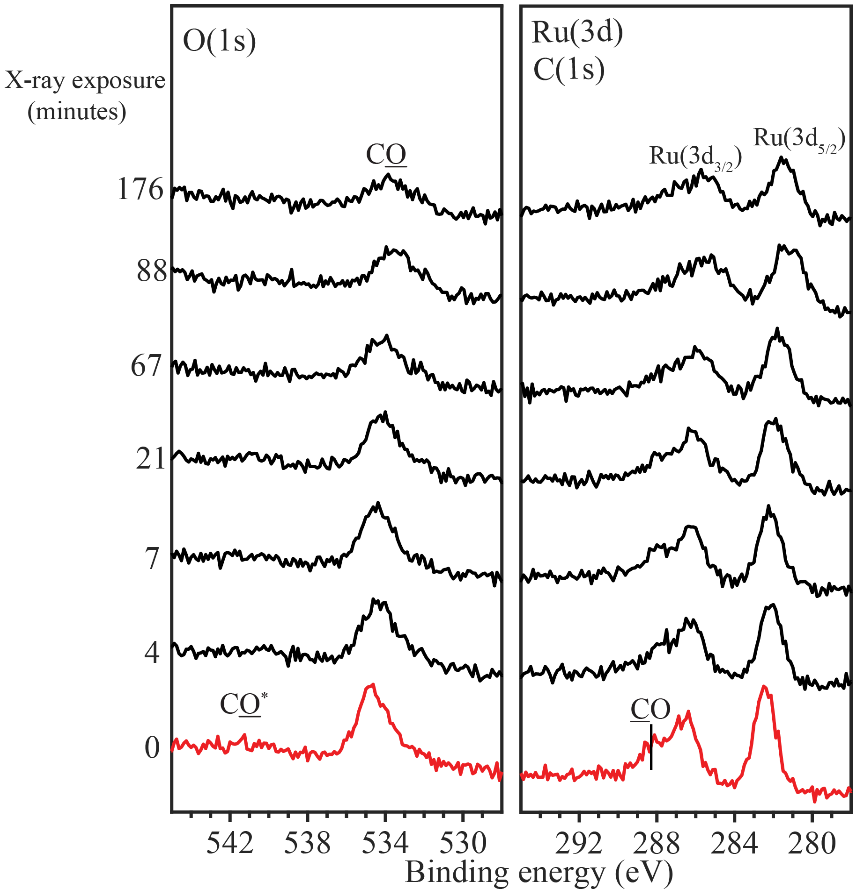

Figure S3: Evolution of the O(1s) and the Ru(3d)/C(1s) XPS regions of $H_2FeRu_3(CO)_{13}$ films exposed to X-rays (Mg Kα 1253.6 eV).

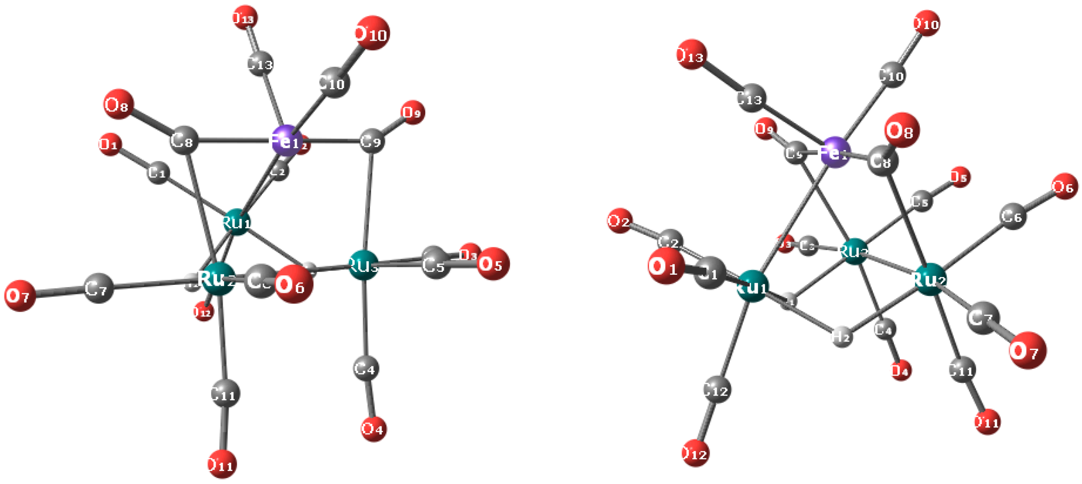

Figure S4: BP86/def2-TZVP optimized neutral and anionic ground state geometry of $H_2FeRu_3(CO)_{13}$ with associated numbers, bond lengths and bond angles given in Table S2 are tabulated using this number schemes.

Table S1: BP86/def2-TZVP optimized Cartesian coordinates of $H_2FeRu_3(CO)_{13}$ neutral molecule and its ground state anion

Neutral:

Ru	-0.273193000	5.357425000	4.114643000
Ru	0.379204000	2.514531000	3.795529000
Ru	0.056881000	4.130721000	1.466407000
Fe	2.112780000	4.468044000	3.162560000
O	0.817848000	5.731427000	6.942025000
O	0.329528000	8.235627000	3.294264000
O	-0.565022000	6.156797000	-0.739957000
O	-2.551897000	2.736814000	0.760548000
O	1.725644000	2.276628000	-0.284319000
O	2.098071000	0.428072000	2.387614000
O	0.427708000	1.143234000	6.529039000
O	2.849179000	3.214998000	5.705331000
O	2.211212000	6.453180000	1.011624000
O	4.493427000	3.218953000	1.983327000
C	0.426399000	5.592590000	5.868353000
C	0.124290000	7.147074000	3.607514000
C	-0.323176000	5.403616000	0.094680000
C	-1.558597000	3.216472000	1.097338000
C	1.100155000	2.971838000	0.388485000
C	1.452482000	1.228010000	2.907535000
C	0.419257000	1.667116000	5.505328000
C	2.283264000	3.481909000	4.714518000
C	1.873597000	5.565151000	1.697569000
C	3.544551000	3.695420000	2.437124000
H	-0.999543000	5.143821000	2.516039000
C	-1.236037000	1.623323000	3.371031000
O	-2.194933000	0.999944000	3.222602000
C	-2.094949000	5.668002000	4.575462000
O	-3.193516000	5.871444000	4.855827000
O	3.511361000	6.661343000	4.490378000

C	2.947575000	5.799043000	3.969411000
H	-0.728665000	3.705992000	4.566086000

Anion:

Ru	-0.222821000	5.419618000	4.149819000
Ru	0.356112000	2.460484000	3.790072000
Ru	0.031123000	4.095071000	1.418717000
Fe	2.156607000	4.475851000	3.170896000
O	0.771097000	5.783579000	7.022698000
O	0.294536000	8.326487000	3.347596000
O	-0.606680000	6.097998000	-0.784456000
O	-2.619125000	2.764134000	0.760225000
O	1.701542000	2.217086000	-0.339679000
O	2.114051000	0.390934000	2.363532000
O	0.380511000	1.080449000	6.504744000
O	2.836836000	3.165799000	5.707213000
O	2.205362000	6.404655000	0.959508000
O	4.600544000	3.316492000	2.045099000
C	0.438892000	5.578622000	5.930468000
C	0.131785000	7.204108000	3.590342000
C	-0.356383000	5.349321000	0.063457000
C	-1.615504000	3.237435000	1.102858000
C	1.138101000	2.936095000	0.375394000
C	1.505168000	1.230410000	2.882989000
C	0.378740000	1.619424000	5.479351000
C	2.291746000	3.463364000	4.708534000
C	1.882290000	5.528582000	1.674939000
C	3.612502000	3.748941000	2.475313000
H	-0.927385000	5.125154000	2.516233000
C	-1.280720000	1.624786000	3.384596000
O	-2.244728000	0.995796000	3.231245000
C	-2.025716000	5.715887000	4.588092000
O	-3.139425000	5.900269000	4.859730000
O	3.449003000	6.709217000	4.517169000
C	2.902071000	5.829846000	3.990880000
H	-0.675250000	3.712673000	4.526340000

Table S2: calculated bond lengths and bond angles of the optimized ground state $H_2FeRu_3(CO)_{13}$ neutral molecule and anion. Geometries are optimized at the BP86/def2-TZVP level of theory. Numbers associated with each atom can be found in Figure S4 here above.

Bond length (Å)	Neutral (calc.)	Anion (calc.)
Fe1-C8	1.84665	1.846
Fe1-C9	1.84582	1.8497
Fe1-C10	1.78134	1.76971
Fe1-C13	1.7662	1.74968
Fe1-Ru1	2.71852	2.74056
Fe1-Ru2	2.68741	2.77252
Fe1-Ru3	2.68653	2.78079
Ru1-C1	1.90269	1.90627
Ru1-C2	1.90211	1.90346
Ru1-C12	1.90463	1.87891
Ru1-H1	1.76883	1.80325
Ru1-H2	1.77158	1.80558
Ru1-Ru2	2.9342	3.03662
Ru1-Ru3	2.93716	3.04595
Ru2-C6	1.89621	1.91212
Ru2-C7	1.9087	1.88721
Ru2-C8	2.32504	2.36559
Ru2-C11	1.893	1.88203
Ru2-H2	1.8002	1.78151
Ru2-Ru3	2.8532	2.89842
Ru3-C3	1.90954	1.88681
Ru3-C4	1.89257	1.88326
Ru3-C5	1.89561	1.91237
Ru3-C9	2.32626	2.3553
Ru3-H1	1.80115	1.78447

Bond angle (degree)	Neutral (calc.)	Anion (calc.)
Fe1-C8-Ru2	79.304	81.328
Fe1-C9-Ru3	79.255	81.845
Fe1-C8-O8	151.26	151.649
Fe1-C9-O9	151.367	151.048
Fe1-C10-O10	178.214	176.982
Fe1-C13-O13	178.972	177.117
C8-Fe1-C9	175.131	175.326
C10-Fe1-C13	97.643	98.717
C8-Fe1-Ru2	58.226	57.509
C9-Fe1-Ru3	58.29	56.974

C10-Fe1-Ru2	107.405	108.872
C10-Fe1-Ru3	107.669	108.96
C13-Fe1-Ru1	90.475	86.332
C10-Fe1-C9	92.22	92.172
C10-Fe1-C8	92.088	92.4
C9-Fe1-C13	88.619	90.102
Fe1-Ru1-C1	92.323	93.497
Fe1-Ru1-C2	91.778	93.217
Fe1-Ru1-C12	167.842	166.102
Fe1-Ru1-H1	90.251	87.674
Fe1-Ru1-H2	90.569	88.079
Fe1-Ru1-Ru3	56.562	57.152
Fe1-Ru1-Ru2	56.619	57.083
Fe1-Ru2-C7	121.345	121.069
Fe1-Ru2-C6	91.018	88.363
Fe1-Ru3-C3	121.476	121.235
Fe1-Ru3-H1	90.59	86.789
Fe1-Ru2-H2	90.957	87.554
Ru1-Ru2-Ru3	60.977	61.705
Ru1-H1-Ru3	110.717	116.203
Ru1-H2-Ru2	110.467	115.673
C1-Ru1-C2	93.016	97.567
C1-Ru1-C12	96.231	95.857
Ru1-C1-O1	178.287	173.604
Ru1-C2-O2	178.193	174.214
Ru1-C12-O12	179.203	179.922
Ru2-Ru3-Ru1	60.873	61.38
C6-Ru2-C7	96.108	97.512
C6-Ru2-C8	90.221	88.006
C6-Ru2-C11	93.355	97.746
C7-Ru2-C8	79.25	80.304
C7-Ru2-C11	90.559	90.309
C6-Ru2-Ru3	93.783	92.41
C7-Ru2-Ru1	109.719	109.301
C2-Ru1-Ru3	97.417	97.389
C1-Ru1-Ru2	97.946	97.225
Ru2-C6-O6	178.628	173.476
Ru2-C7-O7	178.926	178.605
Ru2C8-O8	129.414	126.992
Ru2-C11-O11	173.104	172.297
Ru1-Fe1-Ru2	65.742	66.841
Ru1-Fe1-Ru3	65.829	66.958
Ru2-Fe1-Ru3	64.137	62.923

C3-Ru3-C4	90.69	90.154
C3-Ru3-C5	96.233	97.463
C3-Ru3-C9	79.384	80.501
C3-Ru3-H1	85.811	87.004
C4-Ru3-C9	169.696	169.301
C5-Ru3-C9	90.21	88.467
C5-Ru3-C4	93.645	97.968
C5-Ru3-Ru2	93.194	92.274
C3-Ru3-Ru1	110.299	109.746
Ru3-C3-O3	178.978	178.548
Ru3-C4-O4	173.221	172.179
Ru3-C5-O5	178.923	173.245
Ru3-C9-O9	129.356	127.084

Table S3: calculated threshold (E_{th}) values for the formation of negative ions $[M - Fe(CO)_2]^-$, $[M - Fe(CO)_3]^-$, $[M - Fe(CO)_4]^-$, and $[M - Fe(CO)_4 - n\ CO]^-$ (n = 1 to 7) through dissociative electron attachment to $H_2FeRu_3(CO)_{13}$, these threshold values are compared to corresponding isobaric fragments $[M - nCO]^-$, where n = 4 to 13. Threshold values are derived from single point energy calculation at the PBE0/ma-def2-TZVP level of theory, while the geometry optimizations are carried out at the BP86/def2-TZVP level of theory. ZPE corrections and thermal corrections are included in the calculated threshold values. Appearance energy (AE) values are estimated from the rising part of corresponding ion yield curves. All values are in eV.

Fragment	E_{th} at PBE0/ma-def2-TZVP	Fragment	E_{th} at PBE0/ma-def2-TZVP	AE
$[M - 4CO]^-$	1.69	$[M - Fe(CO)_2]^-$	1.20	0
$[M - 5CO]^-$	3.42	$[M - Fe(CO)_3]^-$	-0.14	0
$[M - 6CO]^-$	5.02	$[M - Fe(CO)_4]^-$	-0.75	0.5±0.1
$[M - 7CO]^-$	7.66	$[M - Fe(CO)_4 - CO]^-$	0.75	1.8±0.2
$[M - 8CO]^-$	8.56	$[M - Fe(CO)_4 - 2CO]^-$	2.82	3.2±0.2
$[M - 9CO]^-$	12.62	$[M - Fe(CO)_4 - 3CO]^-$	3.87	4.4±0.2
$[M - 10CO]^-$	16.30	$[M - Fe(CO)_4 - 4CO]^-$	6.37	7.4±0.2
$[M - 11CO]^-$	15.94	$[M - Fe(CO)_4 - 5CO]^-$	8.59	9±0.2
$[M - 12CO]^-$	19.00	$[M - Fe(CO)_4 - 6CO]^-$	11.00	11.9±0.2
$[M - 13CO]^-$	22.82	$[M - Fe(CO)_4 - 7CO]^-$	13.70	14.6±0.2

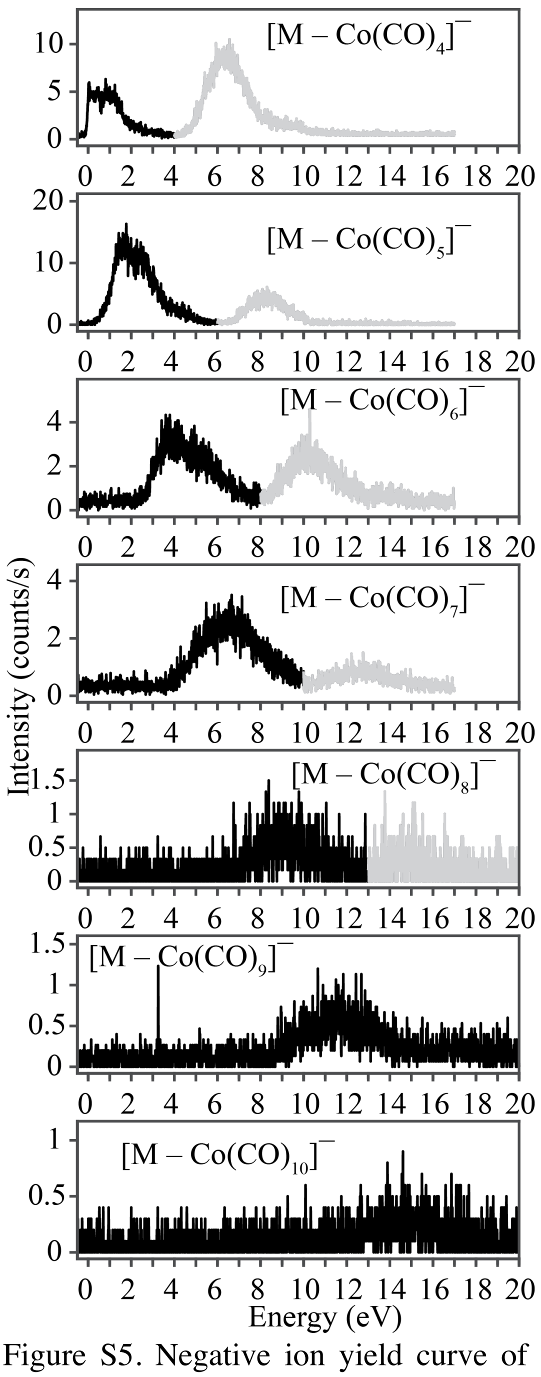

Figure S5. Negative ion yield curve of [M – Co(CO)$_n$]$^-$ (n = 4 to 10) produced through dissociative electron attachment to $HFeCo_3(CO)_{12}$. These spectra are recorded with low mass resolution to attain sufficient signal and the grey shaded contributions are attributed to spill-over from [M – (CO)$_{n+2}$]$^-$.
